# Medicinal Plants from Latin America with Wound Healing Activity: Ethnomedicine, Phytochemistry, Preclinical and Clinical Studies—A Review

**DOI:** 10.3390/ph15091095

**Published:** 2022-08-31

**Authors:** Anuar Salazar-Gómez, Angel Josabad Alonso-Castro

**Affiliations:** 1 Escuela Nacional de Estudios Superiores Unidad León, Universidad Nacional Autónoma de México (ENES-León UNAM), Blvd. UNAM 2011, Guanajuato 37684, Mexico; 2 Departamento de Farmacia, Universidad de Guanajuato, Noria Alta, Colonia Noria Alta Guanajuato, Guanajuato 36250, Mexico

**Keywords:** wound healing, medicinal plant, complementary medicine

## Abstract

Latin America is a multicultural region with ancient traditional medicine. There is extensive knowledge of the use of medicinal plants for wound healing in this region. Nevertheless, many of these medicinal plants lack pharmacological, toxicological, and chemical studies. This review focuses on the ethnomedicinal, phytochemical, and pharmacological (preclinical and clinical) studies of medicinal plants with wound healing activity, from Latin America. An electronic database search was conducted by consulting scientific articles and books. A total of 305 plant species with wound healing activity were recorded, based on traditional medicine. Most medicinal plants used in wound healing in Latin America are topically administered; their methods of preparation are mainly by water infusion from aerial parts. Only thirty-five percent of medicinal plants used in traditional medicine for wound healing have been experimentally validated for their pharmacological effects, and the wound healing activity of five medicinal plants has been studied in clinical trials. In all, 25 compounds (mostly terpenes and flavonoids) have been isolated from medicinal plants with wound healing activity; therefore, extensive work is necessary for a multidisciplinary approach to evaluate the wound healing effects of medicinal plants in Latin America. The mechanism of action of medicinal plants, their toxicological actions on the skin, and their bioactive compounds, have yet to be investigated. This review on the ethnomedicinal, phytochemical, and pharmacological studies, of medicinal plants from Latin America with wound healing activity, offers promising data for further studies, as well as providing new insights into their possible role in wound care.

## 1. Introduction

Skin is the primary barrier that confers protection to the body against physical, biological, and chemical agents. Wounds are breaks, or openings, in the epithelium and can cause physical disability. Untreated wounds might result in hematomas and lacerations. Wound healing could be acute or chronic and focuses on decreasing tissue damage, increasing tissue perfusion and oxygenation, restoring the affected tissue, inducing migration and proliferation of keratinocytes, and promoting angiogenesis. Wound healing is a natural process for repairing and regenerating tissue damage and consists of hemostasis (coagulation), inflammation, proliferation (angiogenesis, granulation, re-epithelialization), and tissue remodeling phases [1,2]. In the hemostasis stage of wound healing, injured blood vessels rapidly constrict and platelet aggregation takes place to clot formation, providing a scaffold for incoming inflammatory cells. Inflammation, the second stage in the wound healing processes, occurs 5–7 h after skin disruption and involves the participation of several pro-inflammatory cytokines, including interleukins (IL)-6 and IL-1β, tumor necrosis factor (TNF)-α, and growth factors such as transforming growth factor-β (TGF-β), that promote vasoconstriction and the activation of neutrophils and macrophages to fight infection and remove debris [3]. At this stage, many opportunistic microorganisms might infect the tissue. Depending on the size of the wound, clot formation and re-epithelization occur 2–4 days after skin disruption and consist of: proliferation of keratinocytes and fibroblasts (attracted by several factors including TGF-β); the synthesis and deposition of collagen and fibronectin in the wounded area; the formation of a new provisional extracellular matrix; and the regeneration of parenchymal and connective tissue cells [1]. Collagen, composed of hydroxyproline, is the main component that provides strength and support to extracellular tissue. In this phase of the wound healing process, the activation of angiogenesis, regulated by the vascular endothelial growth factor (VEGF), occurs. The fibroblast growth factor (FGF), platelet activation factor (PAF), and TGF-β are important elements during this process. During tissue remodeling, epithelial tissue is regenerated, the scar is formed, and leukocyte infiltration and edema are decreased, whereas tensile strength of the skin is increased [1]. Myofibroblasts contract fibers to close wound surfaces. Tissue remodeling can last 1–2 years after skin disruption. Nevertheless, impaired wound healing is affected by age, obesity, diabetes, alcoholism, and other immunity conditions [1].

Latin America is a multicultural region encompassing 43 countries, and was the settlement of many pre-Hispanic civilizations. Their knowledge and culture still remain. This region is the home of over 50 million indigenous people, belonging to 400 ethnic groups [4]. Folk knowledge was incorporated into many pharmacopeias from this region. Latin America has many endemic medicinal plants; however, this region is facing a loss of biodiversity [5]. The migration to rural areas leads to the loss of ethnomedicinal information. In Latin America, using medicinal plants for primary health care is a common practice among the general population, due to the lack/insufficient medical attention, and the lack of economic resources. Around 15% of all plant species in the world have scientific studies about their pharmacology, chemical composition, and toxicology; this indicates the need to carry out more studies on medicinal plants [6]. Ethnopharmacology encompasses the use of traditional knowledge to obtain new drugs from natural resources, with the participation of disciplines such as taxonomy, pharmacology, toxicology, anthropology, biochemistry, and others.

This review highlights the ethnobotany, pharmacology, and phytochemistry of Latin American medicinal plants used for wound healing. Preclinical and clinical studies are analyzed and discussed. Furthermore, formulations containing medicinal herbs are also discussed. The pharmacological treatment of wound healing also includes the oral and topical administration of agents such as analgesics and antibiotics. Nevertheless, these drugs induce several adverse effects; therefore, discovering new agents that accelerate wound healing is highly desirable. This review is essential for further studies on the wound healing activity of medicinal plants from Latin America.

## 2. Results

### 2.1. Ethnomedicinal Information

A total of 305 plant species were recorded with wound healing activity, based on traditional medicine (Table 1). Most of the ethnomedicinal information was gathered from bibliographic sources dated from the last 20 years. Piper genus recorded the highest number (10) of plant species, followed by Croton (six members), and Solanum (five members) genera. The Asteraceae family recorded the highest (42 members) number of plants with wound healing properties, followed by the Fabaceae family (39 members). Only 18% of medicinal plants are orally administered. The main method of preparation is by infusion (36%), followed by decoction (24%), pulverized plant parts (12%), cataplasm (9%), maceration (7%), decoction (6%), and others. The main plant part used are leaves (38%), followed by bark (15%), whole plant (13%), aerial parts (11%), latex (8%), roots (7%), and others. This information shows that medicinal plants used for wound healing in Latin America are topically administered, and the extraction is mainly carried out by infusion with water of aerial parts. The main phytochemical components of the Piper genus are alkaloids, terpenes, lignans, and chalcones [7]. These compounds can be poorly extracted with water. Nevertheless, this genus also contains water-extractable compounds such as flavonoids [7]. Further chemical, pharmacological, and toxicological studies, should be performed with the Piper genus.

### 2.2. Preclinical Wound Healing Research

A total of 108 species (35%) belonging to 50 families had pharmacological evidence of wound healing activity in preclinical and/or clinical studies. Members of the *Asteraceae* and *Fabaceae* families had the highest number of reports on wound healing activity. It is a common trend that, in Latin America, these two botanical families contain a high number of members with medicinal effects [5,8,9,10,11].

Of the 108 plant species with pharmacological activity tested, 66 (61%) had ethnomedicinal information of wound healing activity (Table 1). This finding indicates that 39% of plants (i.e., *Pereskia aculeata* Mill. and *Plinia peruviana* (Poir.) Govaerts) with pharmacological studies on wound healing activity did not consider folk medicinal knowledge. Traditional knowledge is a tool for finding new drugs. For instance, valepotriates (e.g., *valtrate*, *acevaltrate*, and *didro-valtrate*) with sedative actions were isolated from *Valeriana officinalis* L., a medicinal plant used for the folk treatment of anxiety and other mental disorders. On the contrary, drugs such as paclitaxel were not isolated following ethnomedicinal information, due to *Taxus brevifolia* Nutt. was used as a diuretic and bronchodilator agent in traditional medicine [6].

In our literature search, 123 preclinical studies on the wound healing activity of medicinal plants were identified and categorized in extracts with in vitro and in vivo wound healing activity (Table 2 and Table 3), and in vivo wound healing activity of plant extracts with different pharmaceutical formulations (Table 4). A total of 39 plant species were investigated for in vitro wound healing activity; four of these plant species lacked wound healing effects (Table 2). The most frequently used plant parts investigated were leaves, with 24 species, followed by aerial parts (16 species), bark (three species), and fruit (two species), among others. Although aqueous extracts are frequently used in folk medicine, ethanol was the most reported solvent for performing wound healing assays (74%), followed by hexane (33%), methanol (10%), and others. Although infusions are frequently used in folk medicine, ethanolic extracts are used the most for performing wound healing assays. Thus, it would be interesting to validate the activity and safety of the most traditional preparations and identify their bioactive compounds. Compounds such as 3α-hydroxymasticadienoic acid (**1**), 3β, 6β, 16β-trihydroxylup-20(29)-ene (**11**), 1,2 tetradecanediol, 1-hydrogen sulfate, sodium (**17**), and others mentioned in this review (Table 5), are easier to isolate using nonpolar solvents. In this case, the use of solvents (water or ethanol), commonly used in traditional medicine, could not be useful to obtain these kinds of compounds. Some of the studies employed two or more solvents, such as hydroalcoholic solutions. The combination of different solvents could increase the obtention of compounds such as terpenes.

**Figure 1:**
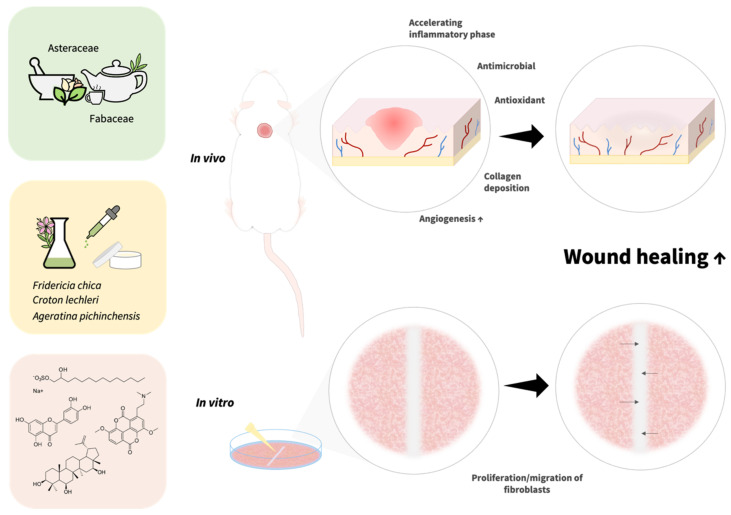
Methods used to evaluate wound healing activity.

The in vitro scratch assay was the method of choice for studying the wound healing activity of these plants, and Swiss 3T3 was the most frequently used cell line, followed by HaCaT and L929. Typically, the scratch assay has been extensively used as a tool for studying cell migration under different experimental conditions (Figure 1). Moreover, this model is effective for the screening and identification of new drugs or active compounds through bioactivity-guided fractionation, and to determine the effective concentration 50 (EC_50_) [64]. Many studies did not report EC_50_ values; this value is important to evaluate the potency of new drugs for wound healing. The calculation of the EC_50_ value is useful for selecting compounds or plant extracts with wound healing activity.

Fibroblast proliferation and collagen synthesis are also two processes to study wound healing under in vitro conditions. Fibroblasts are one the most abundant cells in skin tissue and play an essential role in initiating the proliferative phase and subsequent tissue remodeling during wound recovery. During their proliferation, fibroblasts produce extracellular matrix proteins (fibronectin, hyaluronan, and proteoglycans) and collagen. Thus, decreased fibroblast proliferation could lead to delayed or chronic non-healing wounds [2]. Some plants, such as *Arrabidaea chica* (Bonpl.) B. Verl. (accepted name: *Fridericia chica* (Bonpl.) L.G. Lohmann) and *Mimosa tenuiflora* (Willd.) Poir (Table 2) stimulate fibroblast proliferation, which could be a useful strategy for wound healing.

**Table 2:** In vitro wound healing promoting activity of single medicinal plants used in Latin America.

Botanical Family	Plant Name	Solvent/Plant Part/ Vehicle	Method/Cell Line/ Main Outcome/Wound Healing Activity, %	Reference
Amaranthaceae	*Iresine herbstii* Hook.	n-hexane and ethanol, leaves, aerial parts, and flowers	Scratch assay, Swiss 3T3 mouse fibroblasts, 34.33% (ethanolic extract) and 28.26% (hexanic extract) at 10 μg/mL	[15]
Anacardiaceae	*Schinus molle* L.	n-hexane and ethanol, leaves, aerial parts, and flowers	Scratch assay, Swiss 3T3 mouse fibroblasts, 76.22% (ethanolic extract) and 50.76% (hexanic extract) at 10 μg/mL	[15]
Annonaceae	*Annona crassiflora* Mart.	methanol-acetone-water (7:7:6 *v*/*v*/*v*), seeds	Scratch assay, HaCaT cells, 54% at 1.8 μg/mL and 73% at 3.6 μg/mL	[65]
Apiaceae	*Petroselinum crispum* (Mill.) Fuss	Methanol, leaves	Scratch assay, A549 cells, 59.08% at 500 μg/mL	[66]
Apocynaceae	*Hancornia speciosa* Gomes	96% ethanol, leaves	Scratch assay, primary human gingival fibroblasts, 42.8% at 25 μg/mL	[67]
Arecaceae	*Attalea speciosa* Mart.	Oil, fruit, DMSO (maximal final concentration of 0.5%)	Scratch assay, L929 fibroblasts, concentration dependent manner 3.12–12.5 µg/mL (AUC)	[68]
Asteraceae	*Achyrocline satureioides* (Lam.) DC.	Ethanol, aerial parts	MTT assay, HaCaT cells, stimulated keratinocyte proliferation at 1 μg/mL	[69]
	*Bidens pilosa* L.	Ethanol and decoction, aerial parts	MTT assay, HaCaT cells No effect	[69]
	*Chaptalia nutans* (L.) Pol.	Ethanol and decoction, aerial parts	MTT assay, HaCaT cells No effect	[69]
	*Eupatorium laevigatum* Lam.	Ethanol, leaves, aerial parts, and flowers	Scratch assay, Swiss 3T3 mouse fibroblasts, 30.14% at 10 μg/mL	[15]
	*Galinsoga parviflora* Cav.	n-hexane and ethanol, leaves, aerial parts, and flowers	Scratch assay, Swiss 3T3 mouse fibroblasts, 64.3% (hexanic extract) and 59.83% (ethanolic extract) at 10 μg/mL	[15]
	*Pluchea sagittalis* (Lam.) Cabrera	n-hexane and ethanol, leaves, aerial parts, and flowers	Scratch assay, Swiss 3T3 mouse fibroblasts, 40.66% (hexanic extract) and 43.93% (ethanolic extract) at 10 μg/mL	[15]
	*Wedelia trilobata* (L.) Hitchc. (accepted name: *Sphagneticola trilobata* (L.) Pruski)	95% ethanol, leaves defatted with hexane: ethyl acetate fraction from column chromatography	Scratch assay, L929 fibroblasts, ethyl acetate fraction: 65.7% migration rate in day 1 and 70.5% closure in day 2 at 3 μg/mL	[70]
	*Xanthium cavanillesii* Schouw (accepted name: *Xanthium strumarium* L.)	n-hexane and ethanol, leaves, aerial parts	Scratch assay, Swiss 3T3 mouse fibroblasts, 9.94% (hexanic extract) and 41.17% (ethanolic extract) at 10 μg/mL	[15]
Bignoniaceae	*Arrabidaea chica* (Bonpl.) B. Verl. (accepted name: *Fridericia chica* (Bonpl.) L.G. Lohmann)	Methanol/0.3% citric acid solution, leaves	MTT assay, confluent primary human fibroblasts, growth stimulation (0.25–250 μg/mL). EC_50_ = 30 μg/mL	[71]
Burseraceae	*Bursera morelensis* Ramirez	Hydro-distillation, essential oil, stems, DMEM	Scratch assay, human fibroblasts, ↑ cell migration at 0.01 mg/mL	[48]
Cactaceae	*Pereskia aculeata* Mill.	95% (*v*/*v*) ethanol, leaves, DMEM	Scratch assay, L929 mouse fibroblast cells No effect	[72]
Cordiaceae	*Cordia americana* (L.) Gottschling and J.S. Mill.	Ethanol, Leaves (0.084 μg/mL of rosmarinic acid)	Scratch assay, Swiss 3T3 mouse fibroblasts, 9.8% at 1 μg/mL	[73]
Crassulaceae	*Sedum dendroideum* DC.	n-hexane and ethanol, leaves, aerial parts	Scratch assay, Swiss 3T3 mouse fibroblasts, 37.21% (ethanolic extract) and 27.86% (hexanic extract) at 10 μg/mL	[15]
Fabaceae	*Bauhinia ungulata* L.	Stem wood, extracted successively with hexane and ethanol. Liquid-liquid fractioning, ethyl acetate fraction	Scratch assay, A549 human epithelial cells, ↑ cell migration process and ↓ lesion area to approximately 32.6% and 22.0% at 10 and 100 μg/mL, respectively.	[74]
	*Dipteryx alata* Vogel	95% ethanol, nuts, DMEM/F12 medium	Scratch assay, A549 adenocarcinoma cell line, after 72 h 83% and 67% at 0.5 and 1mg/mL, respectively	[75]
	*Mimosa tenuiflora* (Willd.) Poir.	Water, bark, ethanol-precipitated compounds	MTT, WST, and BrdU incorporation assays, primary natural human fibroblasts (pNHDF), stimulated mitochondrial activity and proliferation at 10 μg/mL	[76]
	*Parapiptadenia rigida* (Benth.) Brenan	Ethanol, bark, fractionation of the extract afforded five known catechin derivatives, DMSO	Scratch assay, Swiss 3T3 mouse fibroblasts, ethanolic extract ~40% at 10 μg/mL	[15]
	*Poincianella pluviosa* (DC.) L.P. Queiroz	Ethanol-water (1:1 *v*/*v*), bark, and fraction	MTT and BrdU incorporation assays, HaCaT cells and human primary dermal fibroblasts (pNHDF). Stimulation of mitochondrial activity and ↑ keratinocyte proliferation	[77]
Hypericaceae	*Hypericum carinatum* Griseb.	n-hexane and cold acetone, aerial parts, phloroglucinol-enriched fractions	Scratch assay, HaCaT cells, ↑ 138.7% cell proliferation at 15 μg/mL	[78]
Loranthaceae	*Struthanthus vulgaris* (Vell.) Mart.	Ethanol, leaves, defatted with hexane	Scratch assay, Swiss 3T3 mouse fibroblasts, 56.2% at 100 μg/mL	[79]
Lythraceae	*Lafoensia pacari* A.St.-Hil.	Hydroethanolic solution (1:10, *w*/*v*), leaves, DMEM medium	Scratch assay, L929 cells, ↑ proliferation/migration of 23.1% and 35.3% at 0.1 and 0.03 μg/mL, respectively	[80]
Malvaceae	*Waltheria douradinha* A. St.-Hil. (syn. *Waltheria communis* A. St.-Hil.)	n-hexane and ethanol, leaves, aerial parts, and flowers	Scratch assay, Swiss 3T3 mouse fibroblasts, 79.70% (hexanic extract) and 54.73% (ethanolic extract) at 10 μg/mL	[15]
Moraceae	*Sorocea houlletiana* Gaudich. (accepted name: *Sorocea guilleminiana* Gaudich.)	Water, leaves, topical, 0.1% DMSO in DMEM	Scratch assay, N3T3 fibroblasts, ~90% proliferation/migration rate at 4 μg/mL	[81]
Myrtaceae	*Eugenia dysenterica* DC.	Essential oil, hydro distillation, leaves	Scratch assay, L929 cells, 100% at 542.2 μg/mL	[82]
	*Plinia peruviana* (Poir.) Govaerts	50% ethanol solution (*v*/*v*), fruit peels	Scratch Assay, L929 cells No effect	[83]
Nyctaginaceae	*Mirabilis jalapa* L.	Ethanol and decoction, aerial parts	MTT assay, HaCaT cells, stimulated keratinocyte proliferation at 25 μg/mL, both extracts	[69]
Onagraceae	*Fuchsia magellanica* Lam.	50% (*v*/*v*) ethanol, leaves	Scratch assay, Swiss 3T3 mouse fibroblasts (120.26%) and HaCaT keratinocytes (114.61%) at 2.5 µg/mL	[84]
Petiveriaceae	*Petiveria alliacea* L.	n-hexane and ethanol, leaves, aerial parts, and flowers	Scratch assay, Swiss 3T3 albino mouse fibroblasts, ethanolic 10.26% at 10 μg/mL	[15]
Piperaceae	*Piper regnellii* (Miq.) C. DC.	Ethanol, leaves, aerial parts, and flowers	Scratch assay, Swiss 3T3 mouse fibroblasts, ethanolic 22.11% at 10 μg/mL	[15]
Plantaginaceae	*Plantago australis* Lam.	Hydroethanolic solution (30% water and 70% ethanol), leaves	Scratch assay, HaCaT cells, 81.06% at 25 µg/mL	[85]
Scrophulariaceae	*Buddleja cordata Kunth*	(CH_2_Cl_2_-methanol) (1:1), Leaves, DMSO- DMEM-F12	Scratch assay, fibroblasts FBH, 33.1% at 50 μg/mL	[86]
Solanaceae	*Brugmansia suaveolens* (Humb. and Bonpl. ex Willd.) Sweet	n-hexane and ethanol, leaves, aerial parts, and flowers	Scratch assay, Swiss 3T3 mouse fibroblasts, hexanic: 26.66% and ethanolic: 9.83% at 10 μg/mL	[15]
	*Solanum diploconos* (Mart.) Bohs	95% ethanol, fresh ripe fruit (peel and pulp with seeds), DMEM	Scratch assay, Murine L929 cells, ↑ fibroblast migration at 1, 10, or 100 μg/mL	[87]

↑ and ↓ denotes an increase or decrease in mentioned variables, respectively; Dulbecco’s Modified Eagle Medium (DMEM).

In all, 39 studies were identified for in vivo wound healing activity of medicinal plants (Table 3). Five of the investigated plants (*Mentzelia cordifolia*, *Muehlenbeckia tamnifolia*, *Mutisia acuminata*, *Spondias mombin*, and *Amphipterygium adstringens*) showed no wound healing activity [88,89]. The leaves (49%), followed by fruit (15%), flowers (15%), and bark (10%), were the most investigated plant parts screened for in vivo wound healing activity. Ethanol was also the most reported solvent for performing wound healing assays (64%), followed by water (62%), methanol (20%), and others. Some of the studies employed two or more solvents for extraction. Since ethanol is the most widely used solvent for the extraction of bioactive compounds such as polyphenols [90], which have generated great interest in wound treatment, the frequent use of this solvent in these assays is not surprising [91]. Consequently, scientists often choose polar solvents, based on the nature of these types of bioactive compounds. The extraction of compounds with wound healing activity, such as terpenes, could be increased with the use of solvents such as dichloromethane. Seven studies have investigated the oral administration of medicinal plants (*Pyrostegia venusta* (Ker Gawl.) Miers, *Plantago australis* Lam., *Persea americana* Mill., *Anacardium occidentale* L., *Coronopus didymus* (L.) Sm., *Chamaesyce hirta* (L.) *Millsp*, and *Cecropia* peltata L.). Interestingly, neither species is related to oral use, based on ethnobotanical claims related to wounds.

**Table 3:** In vivo wound healing promoting activity of single medicinal plants used in Latin America.

Botanical Family	Plant Name	Solvent/Plant Part/ Administration/ Vehicle	Percentage of Wound Healing Activity (Model of Study)	Reference
Anacardiaceae	*Anacardium occidentale* L.	Unripe cashew, fruit pulp, oral, water (juice) 1:1	Swiss mice pretreated daily for 14 days and post-surgery for 21 days, 86% at 0.2 mL on day 14 (excision wound)	[92]
	*Amphipterygium adstringens* (Schltdl.) Standl.	Ethanol-water (70:30), stem bark, topical, propylene glycol:ethanol (90:10)	Wistar rats for 15 days, at 10 mg/wound/day (excision wound) no effect	[89]
	*Schinus terebinthifolia* Raddi	Methanol, leaves, topical, unbuffered physiological saline	Wistar rats for 11 days, 70.88% at 80 mg/mL (excision wound)	[93]
	*Spondias mombin* L.	Fruit, decoction, topical, water	Mice (Strain A) for 2 days, (wound-breaking strength) (incision wound) no effect	[88]
Annonaceae	*Annona squamosa* L.	70% ethanol, leaves, topical	Normal and STZ-induced diabetic Wistar rats for 16 days and 20, respectively; 85% of contraction normal rats at 100 mg/kg (excision wound). For 8 days, ↑ 89% in tensile strength in normal rats and 77% in diabetic rats at 100 mg/kg (incision wound)	[21]
Apocynaceae	*Allamanda cathartica* L.	Water, leaves, topical	Sprague Dawley rats for 14 days, 91.12% at 150 mg/kg/day (excision wound). For 10 days, breaking strength 37.44% at 150 mg/kg/day (incision wound)	[94]
	*Himatanthus drasticus* (Mart.) Plumel	Latex-water (1:1; *v*/*v*), topical, mineral oil	Swiss mice for 14 days, 50 μL on days 3 (37.83%), 7 (69.24%) and 10 (81.56%) (excision wound)	[95]
Asteraceae	*Ageratina pichinchensis* (Kunth) R.M. King and H. Rob.	Water, aerial parts, topical	Sprague-Dawley rats for 8 days, 60% at 16% (*w*/*v*) (incision wound)	[35]
	*Bidens pilosa* L.	90% ethanol, leaves, topical, water	Wistar rats for 9 days, 74.31% at 100 mg/mL (excision wound)	[96]
	*Mutisia acuminata* Ruiz and Pav.	Pulverized stems and leaves, topical, water	Mice (Strain A) for 2 days, (wound-breaking strength) (incision wound) no effect	[88]
	*Vernonia scorpioides* (Lam.) Pers.	Ethanol, flowers and leaves, liquid-liquid fractioning, ethyl acetate fraction	Wistar rats for 7 days, 39.8% at 5 mg/kg (excision wound infected with *Staphylococcus aureus*)	[97]
Basellaceae	*Anredera diffusa* (Moq.) Sperling	Leaves infusion, topical, water	Mice (Strain A) for 2 days, 45.1% wound- breaking strength at 200 mg/mL (incision wound)	[88]
Bignoniaceae	*Arrabidaea chica* (Bonpl.) B. Verl. (accepted name: *Fridericia chica* (Bonpl.) L.G. Lohmann)	Methanol/0.3% citric acid solution, leaves, topical, saline 0.9%	Wistar rats for 10 days, 96% at 100 mg/mL (excision wound)	[71]
	*Pyrostegia venusta* (Ker Gawl.) Miers	Methanol, flowers, oral, DMSO	Wistar rats for 19 days, ~38% on 4th day and ~64% on 7th day at 100 mg/kg (excision wound). For 10 days, ↑ breaking strength at 100 mg/kg (incision wound)	[98]
Brassicaceae	*Coronopus didymus* (L.) Sm.	95% ethanol and aqueous, entire plant, oral, water	Wistar rats, aqueous and ethanolic ~37% tensile strength at 200 mg/kg (incision wound)	[99]
Burseraceae	*Bursera morelensis* Ramírez	Hydro-distillation, essential oil, stems, topical, cosmetic grade mineral oil	CD-1 mice for 10 days, 67.2% at 10% (excision wound); 36% wound healing efficacy at 10% (incision wound)	[48]
Cactaceae	*Hylocereus undatus* (Haw.) Britton and Rose	Water, leaves, flowers, fruit pulp, topical, water	STZ-induced diabetic Wistar rats for 7 days, 90% flowers extract at 0.5% (*w*/*v*); 73% leaves extract at 0.5% (*w*/*v*); 59% fruit pulp extract at 0.5% (*w*/*v*) on day 7 (excision wound). For 7 days, 81% flowers extract at 0.5% (*w*/*v*); 66% leaves extract at 0.5% (*w*/*v*); 52% fruit pulp extract at 0.5% (*w*/*v*) (incision wound; tensile strength)	[100]
	*Opuntia ficus-indica* (L.) Mill.	Water (mucilage extract) and methanol, flowers, topical, methanol extract 10% in glycerol	Wistar rats for 13 days, >98% at 0.5 mg/mm^2^ mucilage extract and methanol extract (excision wound)	[101]
Combretaceae	*Combretum leprosum* Mart.	Ethanol/water solution (8:2 *v*/*v*), leaves, topical, saline.	Swiss albino mice for 12 days, 86.88% at 100 μg/mL (excision wound)	[102]
Cucurbitaceae	*Cucurbita pepo* L. var. Bejaoui	Cold pressed oil, seeds, topical	Wistar rats for 11 days, 91.6% on day 11 at 0.52 μL/mm^2^ (excision wound)	[103]
Equisetaceae	*Chamaesyce hirta* (L.) Millsp previously known as *Euphorbia hirta* L.	95% Ethanol, whole plant, oral	Alloxan induced diabetic Swiss rats for 16 days, wound contraction 35.92, 44.69 and 61.42% at 100, 200 and 400 mg/kg, respectively (excision wound)	[104]
Euphorbiaceae	*Acalypha langiana* Müll. Arg.	Water, leaves, topical	STZ-induced diabetic rats for 7 days. ED_50_ = 0.27% (*w*/*v*) (excision wound), ED_50_ = 0.29% (incision wound), and >ED_50_ = 0.5% (*w*/*v*) (tensile strength).	[105]
	*Croton lechleri* Müll. Arg.	Latex, alkaloid extraction, topical, PBS	Every 12 h for 2 days, 31% at 10% (wound-breaking strength)	[106]
	*Jatropha curcas* L.	Latex, cortex, topical,	Mice (Strain A) for 2 days, 31.1% wound- breaking strength at 100 mg/mL (incision wound)	[88]
	*Jatropha neopauciflora* Pax	Latex, topical	CD-1 mice for 10 days. 100% at 50, 75, and 100% (*w*/*v*) (tensile strength)	[107]
Fabaceae	*Bowdichia virgilioides* Kunth	Water, stem bark, topical, water	Swiss mice for 9 days, 62.5% on day 6 and 91% on day 9 at 10 mg/kg (*w*/*v*) (excision wound); for 9 days, 95.5% at 10 mg/kg (*w*/*v*) (excision wound infected with *S. aureus*)	[108]
	*Caesalpinia ferrea* Mart. ex Tul.	Polysaccharide-rich extract, bark, topical, saline 0.9%	Wistar rats for 21 days, 91.67% at 0.1% on day 10 (excision wound)	[109]
	*Copaifera langsdorffii* Desf.	Oleoresin, bark, topical, 4% Tween 80 in normal saline	Wistar rats for 21 days, 84.05% at 4% on day 9 (excision wound) and for 12 days 99% at 4% on day 5 (incision wound; tensile strength)	[110]
	*Copaifera paupera* (Herzog) Dwyer (syn. *Copaifera langsdorffii* Desf.)	Oleoresin (exudated from the trunk, topical, mineral oil	Alloxan-induced diabetic Swiss Webster mice for 14 days, ~55–60% at 100, 150 or 200 mg/kg from day 7 (excision wound)	[111]
Losaceae	*Mentzelia cordifolia Dombey* ex Urb. and Gilg	Cortex, decoction, topical, water	Mice (Strain A) for 2 days, (wound-breaking strength) (incision wound) no effect	[88]
Lauraceae	*Persea americana* Mill.	Fruit, paste (extract), topical and oral (water)	Sprague Dawley rats for 21 days, ~90% topical and oral at 300 mg/kg/day on day 13 (excision wound); For 10 days, ↑ wet and dry weight of the granulation tissue (dead space wound)	[112]
Martyniaccae	*Martynia annua* L.	Ethanol, defatted dried powdered, leaves, (methanol soluble fraction from chloroform insoluble fraction), topical	Wistar rats for 20 days, 100% at 100% on day 18 (excision wound); for 9 days, ↑ tensile strength (incision wound)	[113]
Meliaceae	*Carapa guianensis* Aubl.	Ethanol, leaves, topical-petroleum jelly, oral-drinking water	Sprague Dawley rats for 15 days, 100% at 250 mg/kg (excision wounds). for 10 days ~51.7% breaking strength at 250 mg/kg (incision wound); for 10 days ↑ wet and dry weight of the granulation tissue at 250 mg/kg (dead space wound)	[114]
Piperaceae	*Peperomia galioides* Kunth	Pulverized stems and leaves, topical, water	Mice (Strain A) for 2 days, 45.9% wound-breaking strength at a dose of 100 mg/mL (incision wound)	[88]
Plantaginaceae	*Plantago australis* Lam.	Hydroethanolic solution (30% water and 70% ethanol), leaves, oral, saline	Wistar rats for 21 days, ~85–95% wound contraction at 500 and 1000 mg/kg, respectively on day 7 (excision wound)	[85]
Polygonaceae	*Muehlenbeckia tamnifolia* (Kunth) Meisn.	Cortex, decoction, topical, water	Mice (Strain A) for 2 days, (wound-breaking strength) (incision wound) no effect	[88]
Solanaceae	*Nicotiana tabacum* L.	Ethanol, Stems defatted previous, topical	Wistar rats for 14 days, 98.7% at 2% (excision wound)	[115]
Urticaceae	*Cecropia peltata* L.	Leaves, aqueous extract (topical and oral), ethanolic extract (topical), water	Sprague Dawley rats for 10 days, aqueous extract 80.71% (topical) and 72.17% (oral) at 150 mg kg^−1^/day on day 11; ethanol extract 79.21% (topical) at 150 mg kg^−1^/day on day 11 (excision wound)	[116]
Verbenaceae	*Lantana camara* L.	Ethanol, leaves, topical	Sprague Dawley rats for 19 days until complete epithelialization, 98% on day 19 at 100 mg/kg daily (excision wound)	[117]

↑ show an increase in the mentioned variables; Streptozotocin: (STZ).

In all, 45 studies were identified for in vivo wound healing activity of medicinal plant extracts, using different pharmaceutical formulations (Table 4). The leaves (50%) were also the most investigated plant parts, followed by fruit (9%), bark (7%), aerial parts (7%), roots (4%), and others. Petroleum jelly and gels were the main ingredients for preparing various formulations, and ethanol extracts, followed by methanol, were the most reported for performing wound healing assays. An important aspect to consider in pharmaceutical formulations is the assessment of their stability, manufacturing processes, and their biological effect in long-term studies. Many plant extracts can suffer degradation of their active compounds through time, and lose or decrease their pharmacological activity.

Excision and incision assays are employed to assess in vivo wound healing (Table 3 and Table 4). Some of these studies reported results using both methods. In four cases, wounds were infected with *Staphylococcus aureus* to assess the effectiveness of antimicrobial activity and faster wound healing rate. Ten studies investigated the efficacy of wound healing-promoting activity in diabetic conditions by simulating the stages of the chronic healing process. Streptozotocin is used for inducing experimental diabetes in rodents. In addition, some medicinal plants, listed in Table 3 and Table 4, were screened for wound healing activity in combination with other in vitro activities, such as anti-inflammatory, antibacterial, and antioxidant effects. Duration of wound healing shows a high variation among studies, ranging from a minimum of 7 to a maximum of 21 days for the excision model and 2 to 30 days for the incision model. The wound repair process occurs in the following order: hemostasis, inflammation, proliferation, and tissue remodeling [2]. The effectiveness of wound healing can be highly dependent on the time and the treatment. The inflammatory phase is crucial in the wound healing process [3]; therefore, a prolonged inflammatory phase could contribute to chronic wound healing. Several in vivo and in vitro studies have demonstrated that medicinal plants and isolated compounds can induce an early inflammation process, which accelerates wound healing [118]. Most of the studies reported wound contraction activity higher than 90% in the first days. For example, topical administration of *F. chica* leaves extract reduces the ulcer area after the 2nd day and healed wounds after 10 days (96%) of treatment in rats [71]; the aqueous extract from *Bowdichia virgilioides* Kunth stem barks induces wound contraction from day 6 and appears to heal wounds in 9 days (91%) in mice [108]. It is possible that these plant extracts induce an early peak of inflammation on day 1, or even earlier. Bardaa et al. [103] demonstrated that extracted oil from *Cucurbita pepo* L. seeds seems to accelerate the hemostasis phase in rats, and contraction was observed from day 3 to day 11 (91.6%) of the experiment. The polysaccharide-rich extract of *Caesalpinia ferrea* Mart. ex Tul. accelerates wound healing by changing the stage of inflammation via modulation of inflammatory mediators [109]. This is an important result, since the main goal in wound management is healing as soon as possible to prevent chronic wound healing. Therefore, in vivo experiments performed up to 11 days would be more relevant to assess the efficacy of wound healing, since wounds in rodents commonly heal between 7–14 days [119]. Particularly in rats, active contraction ceases after 12 days [120].

On the other hand, many studies using the incision wound model found that plant extracts improved wound healing activity by increased wound tensile strength, or breaking strength, suggesting an increase in collagen synthesis and the formation of stable molecular crosslinks to form fibers. In particular, the latex of *Jatropha neopauciflora* Pax and the oleoresin of *Copaifera langsdorffii* Desf. are two promising exudates that increase wound tensile strength by 100% and 99%, respectively. Plants produce different types of exudates, including latexes and oleoresin with a protective capacity against herbivores and phytopathogens [121]. Latex and oleoresin were also two forms of application. This confirmed the potential as a new bioactive chemical resource, not only for wound healing, but also for antimicrobial activity in infected wounds.

Of the 84 reports investigating wound healing activity in vivo, (Table 1 and Table 2), there were a total of 8 irritation studies reported (9.5%). The remaining 76 studies did not report irritation in their results. The studies that reported 90% of wound contraction activity lacked an irritation assessment test as part of the toxicity test. Considering the potential hyper sensibility reaction associated with some plants [122], it is noteworthy to include irritability assays when screening for wound healing properties of medicinal plants and their derivatives. For example, the essential oil from *Lippia sidoides* Cham. (accepted name: *Lippia origanoides* Kunth), commonly found in the Northeast of Brazil, is traditionally used for treating wounds and superficial infections. However, de Oliveira et al. [123] revealed an irritant response to the skin when applied topically in high concentrations, without wound healing activity. Thus, even when no healing wound activity is found during preclinical studies of plants used in traditional medicine, the use of an irritability test can corroborate their safety. A topical application can reduce adverse reactions (e.g., systemic bleeding, duodenal ulcers, electrolyte imbalance, etc.) shown by oral administration. Topical application could reduce pharmacokinetic interactions with other drugs.

**Table 4:** In vivo wound healing promoting activity of formulations based on Latin American medicinal plants.

Botanical Family	Plant Name	Solvent/Plant Part/ Administration/ Vehicle	Percentage of Wound Healing Activity (Model of Study)	Reference
Amaranthaceae	*Alternanthera brasiliana* (L.) Kuntze	Methanol, leaves, topical, petroleum jelly	Immunocompromised Sprague Dawley rats for 7 days, 77.10% at 5% (*w*/*w*) on day 8 (excision wound)	[124]
		Methanol, leaves, topical, petroleum jelly	STZ-induced diabetic Sprague Dawley rats for 7 days, 89.76% at 5% (*w*/*w*) on day 8 (excision wound)	[125]
	*Chenopodium ambrosioides* L.	Ethanol, aerial parts, topical, ointment base	Wistar rats for 19 days, 72.97% and 98.78%, at 5% on days 14 and 19, respectively (excision wound)	[126]
Annonaceae	*Annona crassiflora* Mart.	Ethanol 98%, fruit peel, ethyl acetate and n-butanol fractions (1:1)- polyphenol-enriched fraction, topical, petroleum jelly/lanolin (7:3)	C57BL/6 mice for 7 days, 32%, 38%, and 36% at 2%, 4% and 6%, respectively on day 4. 84% at 2% on day 7 (excision wound)	[127]
	*Annona muricata* L.	Leaves, aqueous semisolid cream, topical	Sprague Dawley rats for 15 days, 69%, and 77% wound closure at 5% and 10%, respectively (excision wound)	[128]
Asteraceae	*Achyrocline alata* (Kunth) DC.	Hydroethanolic solution 70%, flowers, topical, ointment base (lanolin and liquid paraffin)	Swiss mice for 17 days, ~60% at 10% (*w*/*w*) from day 4 (excision wound)	[129]
	*Flaveria trinervia* (Spreng.) C. Mohr	Methanol, leaves, topical, aqueous base (polyethylene glycol and emulsifying wax)	Albino mice for 34 days, 90% at 5% (*w*/*w*) on day 18 (excision wound)	[130]
	*Neurolaena lobata* (L.) Cass.	Ethanol, leaves, topical, petroleum jelly	Sprague Dawley rats for 13 days, 87% at 100 mg/kg/day (excision wound)	[131]
	*Verbesina crocata* (Cav.) Less.	Methanol, aerial parts, topical, Vaseline^®^	CD-1 mice for 14 days, ~33% at 5% *w*/*w* (incision wound; tensile strength)	[132]
	*Vernonia scorpioides* (Lam.) Pers.	Ethanol 96%, leaves, topical, hydrogel Carbopol 2%,	Guinea pigs (H–D) for 30 days, at 50% lacked effects on closure time, but stimulated the regeneration of the new tissue (excision wound)	[133]
Cactaceae	*Pereskia aculeata* Mill.	Methanol and partition with hexane, leaves, topical, gel base: hydroxyethylcellulose (Natrosol 250 HHRs), sodium lauryl sulfate, alcohol, glycerin and methylparaben	C57BL/6 mice for 14 days, 70% hexane fraction at 5% on day 5, 80% both hexane fraction and methanol fraction at 5% on day 7 (excision wound)	[134]
Calophyllaceae	*Calophyllum brasiliense* Cambess.	Ethanol:water (9:1), leaves, topical, non-ionic emulsion: 6% cetostearyl alcohol (*w*/*w*), 5% octyldodecanol (*w*/*w*), 7% mineral oil (*w*/*w*), 12% ethoxylated alcohol stearyl (*w*/*w*), 18% glycerin monoestarate (*w*/*w*), 10% propylene glycol (*w*/*w*), and purified water	Wistar rats for 21 days, 90.67% at 10% (*w*/*w*) on day 14 (excision wound)	[135]
Caryocaraceae	*Caryocar coriaceum* Wittm.	Fixed oil from the seeds, topical, Vaseline^®^ and lanolin (1:2)	Swiss albino mice for 14 days, 96.54% at 12% (*v*/*w*) on day 7 (excision wound)	[136]
Celastraceae	*Maytenus ilicifolia* Mart. ex Reissek	Ethanol 70%, leaves, topical, Vaseline^®^ and lanolin	BALB/c mice for 7 days, after 3 days, 20.8% at 4%; after 7 days 67.9% at 4% (excision wound)	[137]
Convolvulaceae	*Ipomoea batatas* (L.) Lam.	Tuber flour, topical, Beeler’s base	Wistar rats for 10 days, 43% and 75% re-epithelialization process for 4 and 10 days, respectively, at 2.5% (excision wound)	[138]
		1.5 N hydrochloric acid and ethanol 96% (15:85, *v*/*v*), peels of the roots, topical, gel formulation: carbopol, liquid glycerin, propylene glycol, triethanolamine and water	Balb/c mice for 14 days, ~70% at 1% on day 6 (incision wound)	[139]
Equisetaceae	*Chamaesyce hirta* (L.) Millsp (syn. *Euphorbia hirta* L.)	Ethanol 95%, whole plant, topical, hydrophobic ointment: povidone iodine/ethanolic extract of *Euphorbia hirta* whole plants. Petroleum jelly Cetostearyl alcohl PEG 6000 liquid paraffin methyl paraben	Alloxan induced diabetic Swiss rats for 16 days, 32.86 and 36.32% at 5 and 10%, respectively, (excision wound)	[104]
Euphorbiaceae	*Croton zehntneri* Pax and K. Hoffm.	Essential oil, leaves, topical, Pluronic F-127 gels (10% *w*/*w*)	Swiss mice for 15 days, 93% at 20% (excision wound)	[140]
	*Jatropha curcas* L.	Methanol, leaves, topical, petroleum jelly	White albino rats for 21 days, 100% at 15% *w*/*w* (excision wound)	[141]
	*Jatropha gaumeri* Greenm.	Water, latex, topical, glycerin (cream)	Balb/c mice for 20 days, 97.7% at 5% *w*/*w* (tensile strength)	[142]
	*Pedilanthus tithymaloides* (L.) Poit.	Defatted with petroleum ether and extracted with methanol, leaves, topical, ointment base: wool fat 5 g, hard paraffin 5 g, cetostearyl alcohol 5 g, soft white paraffin 85 g	Wistar rats for 18 days, 95.88% at 5% (excision wound), for 10 days 41.17% and 37.6% at 2.5% and 5%, respectively (incision wound; tensile strength); ↑ wet and dry weight of the granulation tissue (dead space wound)	[143]
Fabaceae	*Copaifera langsdorffii* Desf.	Oleoresin and hydroalcoholic, leaves, topical, cream: aqueous phase composed of 75.8% water and 4.0% propylene glycol, and an organic phase contained 17% Lanette cream, 3% hard paraffin, and other components, pH = 6.86.	Wistar rats for 14 days, 95.1% hydroalcoholic at 10% and 94.72% oleoresin at 10% on 14 day (excision wound)	[144]
	*Dipteryx alata* Vogel	Bark extracted with ethanol, topical, cream	C57BL/6 mice (excision wound) for 21 days, at 5%, 10%, and 15% no effect	[145]
	*Mimosa pudica* L.	Distilled water, root, defatted by extracting with pet-ether followed by extraction of methanol, topical ointment base B.P.	Wistar albino rats for 19 days, 87.71% on day 12 and 100% on day 16 at 2% (*w*/*w*) of methanolic extract (excision wound); for 10 days, ↑ tensile strength at 2% (*w*/*w*) (incision wound)	[146]
	*Prosopis juliflora* (Sw.) DC.	Powder, leaves, topical, glycerin	Wistar rats for 21 days, 76% and 85% on day 14 and 21, respectively at 30% (*w*/*w*) (excision wound)	[147]
	*Stryphnodendron adstringens* (Mart.) Coville	Me_2_CO-H_2_O (7:3), stem bark, redissolved in H_2_O and extracted with ethyl acetate (fraction), topical, ointment base (Beeler base)	Wistar rats for 10 days, at 1% (excision wound) no effect	[148]
Lauraceae	*Persea americana* Mill.	Hexane, oil fruit, topical, semisolid formulation petroleum jelly	Wistar rats for 14 days, 100% at 50% on day 13 (excision wound); for 10 days, ~33% at 50% (incision wound; tensile strength)	[149]
		Methanol, seeds, topical, hydrogel: 1% Carbopol base	Wistar albino rats for 20 days, 100% after 16 days at dose 5 and 10% (excision wound infected with *S. aureus*)	[150]
Loganiaceae	*Strychnos pseudoquina* A. St.-Hil.	Selective sequential extraction n-hexane, ethyl acetate, and ethanol/water (9:1, *v*/*v*), stem bark, topical hydroethanolic extract, lanolin	STZ-induced diabetic Wistar rats for 21 days, 90% and 89.9% on day 14 at 5% and 10%, respectively; 98.25% and 98.1% on day 21 at 5% and 10%, respectively; (excision wound)	[151]
		Samples, ethanol 95%, topical, emulsified in lanolin at 5% and 10% (*v*/*v*)	Wistar rats for 21 days, 52.4% and 58.5% on day 7 at 5% and 10%, respectively, (excision wound)	[152]
Loranthaceae	*Struthanthus vulgaris* (Vell.) Mart.	Ethanol, leaves, defatted with hexane, topical, lanolin: petrolatum (3:7)	Wistar rats for 21 days, after 7 and 10 days 72% and 79% at 5%, respectively, (excision wound)	[153]
Lythraceae	*Lafoensia pacari* A.St.-Hil.	Hydroethanolic solution (1:10, *w*/*v*), leaves, topical, 2% propylene glycol and incorporated into Sepigel^®^	Wistar rats for 24 days, 57.5% at 30 mg/g on day 6 (excision wound); for 9 days, ~42% at 100 mg/g (incision wound; tensile strength)	[80]
Martyniaccae	*Martynia annua* L.	Ethanol, defatted dried powdered, leaves, topical, ointment base B.P. (ointment)	STZ-induced diabetic Wistar albino rats for 20 days, 100% methanol soluble fraction (from chloroform insoluble fraction) at 5% (*w*/*w*) on day 18 (excision wound)	[154]
Moraceae	*Dorstenia drakena* L.	Water, rhizome, topical, petroleum jelly (ointment)	Wistar rats for 30 days. 25% at 20% (*w*/*w*) (incision wound)	[155]
	*Sorocea houlletiana* Gaudich. (accepted name: *Sorocea guilleminiana* Gaudich.)	Water, leaves, topical, 1% propylene glycol and incorporated into Polawax™ NF (12%) (from Croda International Plc, Goole, East Yorkshire, UK), mineral oil (5%), propylene glycol (3%), volatile silicone (2%), EDTA (0.1%), methylparaben (0.1%), and propylparaben (0.05%) in distilled water. (cream)	Wistar rats for 23 days, 82.66% on day 7 and 90.08% on day 9 at 2 mg/g (excision wound); for 9 days at doses of 2 mg/g (45.73%) and 50 mg/g (35.27%) (incision wound; tensile strength)	[81]
Orchidaceae	*Prosthechea michuacana* (Lex.) W.E. Higgins	Hexane, bulbs, 2% (*v*/*v*) Tween-80 and simple ointment bases (ointment)	Wistar albino rats for 18 days, 99.2% at 50% (*v*/*v*) on day 16 (excision wound); for 7 days, ↑ tensile strength at 50% (incision wound)	[156]
Passifloraceae	*Passiflora edulis* Sims	Ethanol/water (7:3), leaves, liquid/liquid partition (butanol fraction, 6.1% isorientin), topical, chitosan hydrogel	Alloxan-induced diabetic Wistar rats for 14 days, 46.28% on day 2 and 96.26% on day 14 at 0.1% (*w*/*v*) (excision wound)	[157]
Plantaginaceae	*Plantago australis* Lam.	70% ethanol and 30% water, leaves, topical, lanolin:Vaseline^®^ (2:3).	Wistar rats for 14 days, ~80% after 7 days at 4% (*w*/*w*) (excision wound)	[158]
Rubiaceae	*Hamelia patens* Jacq.	Ethanol, aerial parts, topical, petroleum jelly (ointment)	Sprague-Dawley rats for 12 days, 16% (double incision; breaking strength) at 10% (*w*/*w*)	[159]
Sapindaceae	*Dodonaea viscosa* Jacq.	Ethanol, leaves, topical, petroleum jelly, lanoline, and paraffin (ointment)	Balb/c mice for 7 days. ED_50_ = 5% (*v*/*v*) (tensile strength)	[160]
Solanaceae	*Capsicum annuum* L.	Methanol, fruit, topical, 1% Carbopol (gel)	Wistar rats for 20 days, 100% on day 16 at 5% and 10% (excision wound infected with *S. aureus*)	[161]
	*Physalis angulata* L.	70% methanol, leaves, topical, aqueous cream (British Pharmacopoeia)	Wistar rats for 15 days, ~95% at 2.5, 5 and 10% from day 10 (excision wound)	[162]
	*Solanum diploconos* (Mart.) Bohs	95% ethanol, fresh ripe fruit (peel and pulp with seeds), topical, semisolid formulation.	Swiss mice for 7 days, reduction (~35%) at 1% on day 7 (excision wound)	[87]
Vernenaceae	*Lippia gracilis* Schauer	Essential oil, leaves, topical, 70% petroleum jelly and 30% anhydrous lanolin	Wistar rats for 21 days, ~72.5% at 10% from day 7 (excision wound)	[163]
	*Lippia sidoides* Cham. (accepted name: *Lippia origanoides* Kunth)	Essential oil, topical, Vaseline^®^ and Lanolin (1:2)	Wistar rats for 21 days, at 6% and 12% (*v*/*w*) (excision wound) no effect	[123]
Ximeniaceae	*Ximenia americana* L.	70% hydroalcoholic, bark and wood, topical, Lanette base	Wistar rats for 14 days, 71% and 86.9% on day 7 and 14, respectively, at 10% (excision wound)	[164]

↑ show increase in mentioned variables.

In many cases, phytochemical studies were carried out to identify the main components of the extracts using analytical techniques such as HPLC, HPLC-ESI-MS/MS, NMR, and GC-MS, which are currently used for chemical standardization of plant extracts. These techniques require specific knowledge of analytical methods and special training. Among the identified compounds, phenolic compounds such as chlorogenic acid, catechin, and quercetin derivatives, are the most reported. Five of these investigations have pursued the standardization of crude plant extracts. Considering that natural ingredients, including plant extracts, are becoming more popular in modern skin care formulations [165], appropriate standardization could improve efficiency and ensure their safety and quality control. It is known that, in some cases, chemical-standardized plant extracts displayed better activities than isolated compounds. This can be attributed to the synergistic effects among compounds, rather than one compound responsible for biological activity.

**Figure 2:**
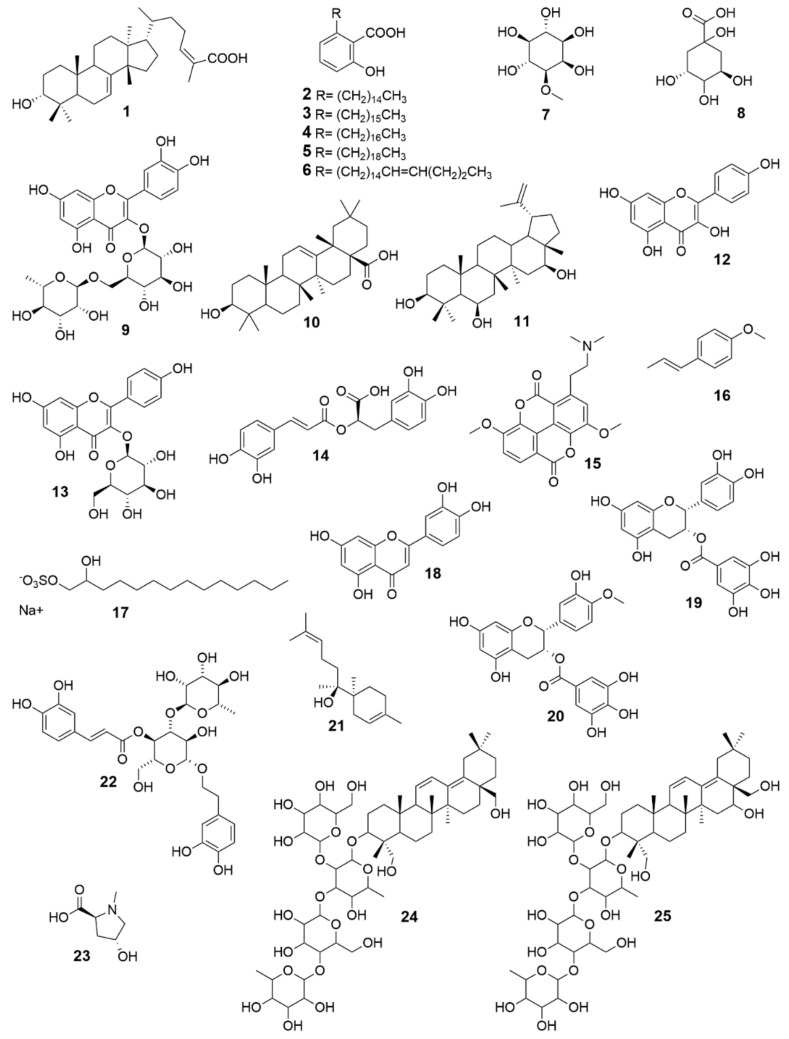
Chemical structures of compounds **1**–**25**.

In all, 15 studies reported the isolation of bioactive compounds (Table 5). A total of 25 compounds, mostly belonging to the terpenoid and flavonoid classes, were isolated from 16 plants and investigated for wound healing activity, using various preclinical models (Table 5, Figure 2). Although it is sometimes difficult to investigate the effect of isolated compounds, due to the small quantities obtained, many of the isolated compounds reported here have been tested in rodent models. To perform and improve these experiments, the authors reported the use of the commercial form of some compounds, such as oleanolic acid (**10**) [44], *trans*-anethole (**16**) [140], and (+)-*epi*-α-bisabolol (**21**) [166]. The isolation of new compounds requires more time to elucidate the chemical structure; this is a challenge that faces many scientific groups. The molecular mechanisms of action underlying the wound healing effect of most of the isolated compounds listed in this review are yet to be clarified. However, some representative compounds generally displayed their wound healing activity by modulation of inflammatory mediators, migration and/or proliferation of fibroblasts, antioxidant effect, enhanced angiogenesis, and/or increased collagen deposition (Figure 1).

**Table 5:** Wound healing effect of bioactive compounds isolated from Latin American medicinal plants (structures illustrated in Figure 2).

No.	Compound	Plant Source	Formulation or Vehicle	Percentage of Wound/ Healing Activity (Model of Study)	Reference
**1**	3α-hydroxymasticadienoic acid	*Amphipterygium adstringens* (Schltdl.) Standl.	Topical, propylene glycol:ethanol (90:10)	Wistar rats for 15 days, ~80% on day 7 at 300 μg/wound/day (excision wound)	[89]
**2–6**	Anacardic acids	*Amphipterygium adstringens* (Schltdl.) Standl.	Topical, propylene glycol:ethanol (90:10)	Wistar rats for 15 days, ~80% on day 7 at 300 μg/wound/day (excision wound)	[89]
**7**	Bornesitol	*Hancornia speciosa* Gomes	DMSO, 0.5%	Scratch assay, primary human gingival fibroblasts, 80.8% at 50 μM (9.71 μg/mL)	[67]
**8**	Quinic acid	*Hancornia speciosa* Gomes	DMSO, 0.5%	Scratch assay, primary human gingival fibroblasts, 69.1% at 50 μM (9.61 μg/mL)	[67]
**9**	Rutin	*Hancornia speciosa* Gomes	DMSO, 0.5%	Scratch assay, primary human gingival fibroblasts, 39.6% at 50 μM (30.53 μg/mL)	[67]
**10**	Oleanolic acid	*Anredera diffusa* (Moq.) Sperling	Topical, DMSO	Mice (strain A) for 2 days, 42.9% at 12.5 mg/mL (incision wound; tensile strength)	[44]
**11**	3β, 6β, 16β-trihydroxylup-20(29)-ene	*Combretum leprosum* Mart.	Topical, saline	Swiss albino mice for 12 days, 99.65% at 100 μg/mL (excision wound)	[102]
**12**	Kaempferol	*Ipomoea carnea Jacq.*	Topical	Wistar rats for 14 days, 91.1% at 200 mg/kg body weight/day (excision wound) 22.6% at 200 mg/kg body weight/day (incision wound; breaking strength)	[167]
**13**	Kaempferol 3-*O*-α-D-glucoside	*Ipomoea carnea Jacq.*	Topical	Wistar rats for 14 days, 90.2% at 200 mg/kg body weight/day (excision wound); 38.3% at 200 mg/kg body weight/day (incision wound; breaking strength)	[167]
**14**	Rosmarinic acid	*Cordia americana* (L.) Gottschling and J.S. Mill.	DMSO, 0.5%	Scratch assay, Swiss 3T3 albino mouse fibroblasts cells, 11.8% at 10 μg/mL	[73]
**15**	Taspine hydrochloride	*Croton lechleri* Müll. Arg.	Topical, PBS	Mice (strain A) every 12 h for 2 days, wound-breaking strength, 58.2% at a dose of 0.1 mg/mL (incision wound) ED_50_ = 0.375 mg/kg	[106]
			PBS	Scratch assay, human foreskin fibroblast, 27 ± 1.83 no. cells/cm at 2 ng/mL	[106]
**16**	Trans-anethole	*Croton zehntneri* Pax and K. Hoffm.	Topical, Pluronic F-127 gels (10% *w*/*w*)	Swiss mice for 15 days, 90% at a dose of 20% (excision wound)	[140]
**17**	1,2 tetradecanediol, 1-(hydrogen sulfate), sodium sal	*Pedilanthus tithymaloides* (L.) Poit.	Topical, ointment base: wool fat 5 g, hard paraffin 5 g, cetostearyl alcohol 5 g, soft white paraffin 85 g	Wistar rats for 18 days, 100% at a dose of 0.25% *w*/*w* (excision wound); for 10 days 50.8% at 0.25% *w*/*w* (incision wound; breaking strength)	[143]
**18**	2-(3,4-dihydroxy-phenyl)-5,7-dihydroxy-chromen-4-one	*Pedilanthus tithymaloides* (L.) Poit.	Topical, ointment base: wool fat 5 g, hard paraffin 5 g, cetostearyl alcohol 5 g, soft white paraffin 85 g	Wistar rats for 18 days, 100% at a dose of 0.25% *w*/*w* (excision wound); for 10 days 51.8% at 0.25% *w*/*w* (incision wound; breaking strength)	[143]
		*Martynia annua* L.	Topical, ointment base B.P. (ointment)	STZ-induced diabetic Wistar albino rats for 20 days, 87.67% at 0.5% *w*/*w* on day 18 (excision wound)	[154]
**19**	Epicatechin-3-*O*-gallate	*Parapiptadenia rigida* (Benth.) Brenan	DMSO	Scratch assay, Swiss 3T3 albino mouse fibroblasts, ~58% increased cell numbers at 1 μM	[168]
**20**	4′-*O*-methylepicatechin-3-*O*-gallate	*Parapiptadenia rigida* (Benth.) Brenan	DMSO	Scratch assay, Swiss 3T3 albino mouse fibroblasts, ~60% increased cell numbers at 1 μM	[168]
**21**	(+)-*epi*-α-bisabolol Some authors referred (+)-anymol	*Peperomia galioides* Kunth	Topical	Mice (strain A) every 12 h for 2 days, wound-breaking strength, 62% using 4 mg per mouse (incision wound), ED_50_ = 155 μg/g	[166]
**22**	Verbascoside	*Plantago australis* Lam.	DMSO	Scratch assay, HaCaT cells, 58.7% and 57.77% at 5 and 10 µg/mL, respectively	[85]
**23**	*N*-Methyl-(2*S*,4*R*)-*trans*- 4-Hydroxy-L-Proline	*Sideroxylon obtusifolium* (Humb. ex Roem. and Schult.) T.D. Penn.	Topical, 1% carboxyvinyl polymer, 5% glycerin, and 1% polypropy- lene glycol. Methylparaben (0.15%) and propilparaben (0.05%) (gel)	Swiss mice for 12 days, reductions around 58% at 3% and 10% (excision wound)	[169]
**24**	3-*O*-α-L-rhamnopyranosyl-(1→4)-β-D-glucopyranosyl- (1→3)-[β-D-glucopyranosyl-(1→2)]-β-D-fucopyranosyl- 23,28-dihydroxyoleane-11,13(18)-diene	*Buddleja scordioides* Kunth	Topical, water	STZ-induced diabetic Wistar rats for 7 days, 70% compound **1** at 0.5% (excision wound), 75% compound **1** at 0.5% (incision wound)	[170]
**25**	3-O-α-L-rhamnopyranosyl-(1→4)-β-D-glucopyranosyl-(1→3)-[β-D-glucopyranosyl-(1→2)]-β-D-fucopyranosyl-16,23,28-trihydroxyoleane-11,13(18)-diene	*Buddleja scordioides* Kunth	Topical, water	STZ-induced diabetic Wistar rats for 7 days, 81% compound **2** at 0.5% (excision wound), 85% compound **2** at 0.5%, (incision wound)	[170]

### 2.3. Clinical Wound Healing Research

Despite some promising preclinical results, clinical research aiming to study the wound healing activity of Latin American plants is still limited, and only seven clinical trials were found in the search (Table 6), mostly double-blind randomized controlled trials. Only one study [171] was carried out in a long-term period (1 year). Clinical assays should also consider chronic wounds, which affect the patient’s quality of life. The treatment of chronic wounds is expensive, and ineffective in some cases, and some drugs can induce side effects that may lead to many patients abandoning their treatment [172]. All studies are phase 1 and include a low number of participants (<100). Clinical studies are expensive, but it is necessary to continue the evaluation of plant extracts cited in the table in phase 2 and 3 assays.

We noted that three studies employed extracts for chronic venous leg ulcers, and involved less than 50 subjects. Among these, *Mimosa tenuiflora* (Willd.) Poir. reduces ulcer size by 93% after the 8th treatment week [173]. Individuals living with venous leg ulcers suffer from symptoms such as pain, odor, exudate, and swelling, combined with restricted mobility, resulting from compression therapy that negatively affects their quality of life [174]. Medicinal plants are promising options for treating venous ulcers [175]. Thus, this is a potential field to evaluate the effect of those plants with important wound healing activity. It is crucial to note that all the plant species used in the clinical trials listed in Table 6 have reported traditional use related to wound healing. These features emphasize the importance of how ethnomedical information provides significant advantages for identifying which plants are most likely to be useful in clinical trials.

**Table 6:** Clinical trials with plants presenting wound healing activity.

Plant Name	Solvent/Plant Part/ Administration/ Vehicle	Study Design	Dose, Duration, and Patient Type	Main Outcome	Adverse Effect	Reference
*Ageratina pichinchensis* (Kunth) R.M. King and H. Rob.	Hexane-ethyl acetate extract, aerial parts, topical, carboxymethyl cellulose (AKUCELL 2201)	Double-blind, prospective, and randomized study	- Standardized extract (0.76% encecalin) - 1-year - Patients with chronic venous leg ulcers (*n* = 17)	Ulcer size reduction 79.1% in the second treatment month. Heal completely 8 months	Tolerability	[171]
	Hexane-ethyl acetate extract, aerial parts, topical, cold cream	Randomized, double-blind, controlled pilot study	- Standardized extract [in 7-O-(β-D-glucopyranosyl)-galactin content] 5% - 24 weeks - Patients with diabetic foot ulcer (*n* = 18)	At week 8 90.78% wound size reduction	No adverse effects	[176]
*Croton lechleri* Müll. Arg.	80% ethanol, bark latex, topical, 10% cetyl alcohol, 7% isopropylmeristat, and 21% Vaseline (cream base); span 20 and tween 80 (emulsifying agent) parabens (preservative); and propylene glycol (humectant)	Double-blind, placebo-controlled, randomized	- 15% ethanolic extract - 20 days - Patients referred for skin tag removal (*n* = 60)	At day 14 95.73% of wound healing	Not mentioned	[177]
*Rhizophora mangle* L.	Aqueous extract, bark	Single blind, randomized and comparative with an antiseptic	- Tannin content higher than 12 mg/mL (condensed tannins 80% and hydrolysable tannins 20%) - 6 weeks - Patients with open wounds from surgical intervention of pilonidal cyst or fistula (*n* = 12)	At day 30, 32% of wound healing	No adverse effects	[178]
*Mimosa tenuiflora* (Willd.) Poir.	Ethanol, cortex, topical, each 100 g of the hydrogel had Carbopol 940 (0.75 g), triethanolamine (0.85 g), *M. tenuiflora* extract (5 g), ethanol (10 g), propylene glycol (16.5 g), methylparaben (0.05 g) and vehicle (100 g).	Randomized, placebo-controlled, double blind	- Standardized hydrogel with 10 mg/g of mimonoside A and 0.5 mg/g of mimonoside B - 8 weeks - Patients with venous leg ulcers (*n* = 22)	No effect	Pain and burning in four patients	[179]
	Ethanol, Bark, topical, polyethylene glycol (PEG-200) and incorporated into Carbopol^®^ 940	Randomized, double-blind, placebo-controlled clinical trial	- Standardized in its polyphenolic content (1.8 g tannins/100 g hydrogel) - 12 weeks - Patients with venous leg ulcers (*n* = 20)	93% ulcer-size reduction at the 8th treatment week	No adverse effects	[173]
*Achillea millefolium* L.	90% ethanol, topical, Vaseline	Double-blind clinical trial study	- 5% - 14 days - Episiotomy wound healing in primiparous women (*n* = 32)	Reduce perineal pain level, redness, edema, and ecchymosis	Not mentioned	[180]

### 2.4. Mechanism of Action

The molecular mechanisms underlying the beneficial effects of some of the plants listed here are associated with different stages of the wound healing process. The common wound healing mechanism of action involves the modulation of inflammatory mediators (i.e., TNF-α, IL-1, and IL-6), either by inducing the production of pro-inflammatory cytokines in the early wound healing process, or by reducing the expression of pro-inflammatory cytokines after 48 h. Simultaneously, the accumulation of anti-inflammatory cytokines and growth factors (i.e., IL-10 and TGF-β) contribute to successful wound closure.

TNF-α, IL-6, and IL-1β, are closely involved in the early wound healing process [181,182], with a gradual decline thereafter. A subtle balance of these pro-inflammatory cytokines is crucial for coordinating cellular processes during wound healing, since overexpression, or a very low reduction, can generate an unfavorable environment and alter the outcome of wound repair. For example, a persistently high level of TNF-α is associated with impaired healing leading to a decrease in collagen production, while regulated production of IL-6 can induce collagen deposition in wound sites [182].

The oral administration of 100 mg/kg methanolic extract of *Pyrostegia venusta* flowers increased the levels of IL-10, with the subsequent decrease in the serum levels of TNF-α and IL-6 after 48 h of treatment in Wistar rats, with excision wound, without affecting collagen formation and hydroxyproline production [98]. IL-10 can limit tissue damage caused by inflammation by decreasing the levels of pro-inflammatory mediators and providing protection against further tissue damage, which helps wound closure, with less scar formation [1,3].

In alloxan-diabetic mice, the topical treatment with 200 mg/kg oleoresin from *Copaifera paupera* decreased the TNF-α levels and increased the levels of IL-10 after 7 days of treatment, whereas the levels of MCP-1 (monocyte chemoattractant protein-1) were decreased after 10 days of treatment [111]. A study indicated that low expression of MCP-1 in *db*/*db* mice liver could explain the constant infections under diabetic conditions, and the same study suggested that using MCP-1 in the early stages could promote the healing of diabetic wounds [183].

On the other hand, topical application of formulations containing 10% oleoresin and 10% hydroalcoholic extract of leaves from *C. langsdorffii* reduced the levels of TNF-α, IL-1β, and IL-6 levels, and increased the IL-10 levels after 3 days in skin biopsies [144]. In addition to IL-10, *C. langsdorffii* increased the FGF-2 and TGF-β (growth factors) gene expression, which are closely involved in granulation tissue formation, re-epithelialization, matrix formation, and remodeling. The wound healing effect of *Strychnos pseudoquina* A. St.-Hil. is also associated with the increase of IL-10 and TGF-β levels, as well as cellularity stimulation, collagen, elastic fibers deposition, and attenuation of oxidative damage in scar tissue [151,152].

Another important mechanism proposed for the wound healing activity of *C. langsdorffii* is the regulation of MMP-2, and MMP-9 expression: two matrix metalloproteinases that contribute to the advancement of the proliferative stage, including migration of fibroblasts and keratinocytes, angiogenesis, and re-epithelialization. These observations suggest the influence of *C. langsdorffii* creams stimulating the wound re-epithelialization mechanism [144]. Similarly, de Moura et al. [137] demonstrated that topical treatment with the hydroalcoholic extract of *Maytenus ilicifolia* Mart. ex Reissek leaves contributes to the advancement of the proliferative stage, probably by increasing the activity of MMP-9.

Nitric oxide (NO) also plays a central role in the regulation of homeostasis, inflammation, and antimicrobial action, in the wound healing process. NO is produced at higher levels by iNOS (inducible nitric oxide synthase) during the inflammatory phase, and modulation of this production has been seen as an attractive solution to impaired wound healing [184]. The wound healing effect of the three plants listed here is associated with NO regulation.

The reduction of TNF-α and IL-1β levels, and MPO (myeloperoxidase activity) accompanying the up-regulation of TGF-β, iNOS, and NO production, was observed in the first days after wound induction in rat topical treatment with polysaccharide-rich extract of *Caesalpinia ferrea* stem barks [109]. The authors suggested that the reduced polymorphonuclear influx and expression/levels of cytokines (TNF-α, IL-1β), paralleled with the increased levels of antinociceptive mediators (iNOS, NO) in the first days after the wound induction, make the polysaccharide-rich extract of *C. ferrea* stem barks an ideal candidate for the treatment of inflammatory and painful cutaneous wounds.

Topical application of a gel containing 10% *N*-Methyl-(2*S*,4*R*)-*trans*-4-Hydroxy-L-Proline (**23**), an L-proline derivative isolated from *Sideroxylon obtusifolium* (Humb. ex Roem. and Schult.) T.D. Penn. leaves, improves the wound healing process by upregulating iNOS and COX-2 (cyclooxygenase-2) activities in the second day after wound induction [169]. Interestingly, other proline derivatives are present in plant extracts with anti-inflammatory and antinociceptive effects [185,186]. Due to the close relationship in the L-proline derivatives structures, (**23**) could be effective in pain management and wound care.

Although NO is produced at higher levels by iNOS during the inflammatory phase [184], the role of this molecule in wound healing could be controversial, according to other studies. For example, topical administration of 5% *Struthanthus vulgaris* (Vell.) Mart. ointment reduces NO production in the early stages of healing, as well as altering the release of TNF-α, IL-1α, IL-10, and TGF-β [153]. Looking forward, future research on the wound healing effect of medicinal plants should also focus on NO production, and its related biochemical mechanisms, since the exact mechanisms of action are poorly understood.

Another component of cell signaling involved in wound healing is the activation of the mitogen-activated protein kinase (MAPK) signaling pathway, particularly extracellular signal-regulated kinase (ERK) 1/2 and p38 MAPK. Both ERK1/2 and p38 are activated by tissue wounding facilitating wound closure. ERK 1/2 pathway activation is associated with the mediation of proliferation, differentiation, and migration, of various cell types [187]. The hydroethanolic leaf extract of *Lafoensia pacari* A.St.-Hil. increases the p-ERK1/2 expression in L929 fibroblasts. The specific mechanism of the hydroethanolic leaf extract of *L. pacari* bioactivity is not completely characterized, but the experimental model used in this work allowed the authors to suggest that this plant might exert cell proliferation during wound healing [80].

### 2.5. Future Considerations

Limited irritability studies are currently available, suggesting that detailed toxicity assays are still required for different medicinal plant extracts, especially for those with significant wound healing activity. Several compounds (i.e., 2-(3,4-dihydroxy-phenyl)-5,7-dihydroxy-chromen-4-one, 1,2 tetradecanediol, Kaempferol 3-O-α-D-glucoside, and others) showed wound healing activity that could be considered for candidates in clinical trials. Special attention should be given to plant extracts such as *Croton lechleri*, *Ageratina pichinchensis*, and others, which showed wound healing activity without adverse reactions in clinical trials. All plants reported with clinical wound healing activity have an ethnomedicinal use related to healing wounds. Thus, traditional knowledge is a useful tool to obtain plants as candidates for clinical evaluation.

Latin American countries face economic and health problems due to the high prevalence of diabetes, and the elderly population is expected to increase in the next two decades [188]. The wound healing activity of plant extracts and compounds should also be evaluated in diabetes-induced in vivo models, where a risk of infection can occur. Access to primary healthcare systems is difficult in this region; therefore, people living in rural areas still depend on traditional medicine. Plants with wound healing activity evaluated in clinical trials could be incorporated into national health systems, as an alternative therapy, in case of lacking allopathic medicine in rural areas.

## 3. Materials and Methods

An electronic database search was conducted using ScienceDirect, PubMed, SciFinder, SciELO, and Google Scholar from January to June 2022. The search included the following keywords: “American”, “Latin American”, “medicinal plants”, “wound healing”, “ethnopharmacological”, “ethnomedicinal”, and “ethnobotanical”. Ethnobotanical information from books was also considered for this review. Only scientific articles published in English, Spanish, or Portuguese, were contemplated. In ethnobotanical studies, only information presenting complete data on the way of preparation, route of administration, plant part used, and the scientific name of the plant species was considered. Only preclinical and clinical studies regarding the single use of a medicinal plant were considered. The study design, dose, duration, patient type, the main outcome, and adverse effects of the herbal treatment, were collected from clinical trials. Scientific works without information on the percentage of wound contraction, tensile strength, or breaking strength, were excluded. In some cases, percentages of wound healing activity were calculated from the information presented in the scientific article. No pharmaceutical formulations based on nanotechnology were contemplated. For each species, the accepted botanical names were validated and updated, if necessary, by consulting the Missouri Botanical Garden (http://tropicos.org/, accessed on 12 May 2022). The chemical structures were drawn using the ChemDraw Ultra 12.0 software.

## 4. Conclusions

In Latin America, there is extensive folk knowledge of medicinal plants for wound healing. This practice is important for primary health care, especially in rural areas. Medicinal plants from Latin America are a source of compounds with wound healing activity. A few plant species (22%) have been scientifically validated to support ethnomedical use, and demonstrate their efficacy. Since 65% of medicinal plants with empirical wound healing activity are yet to be studied, extensive work is necessary for a multidisciplinary approach to evaluate the wound healing effects of medicinal plants in Latin America.

Only two formulations containing plant extracts were evaluated for wound healing activity in clinical trials. The pharmaceutical formulation requires the evaluation of their manufacture and stability, for assessing their efficacy in clinical trials.

Several reports have described the wound healing potential of plant extracts, or their isolated compounds, but their mechanism of action are yet to be explained. The mechanism of action could help to prepare combinations of compounds with different mechanisms of action for inducing synergistic actions. It is necessary to carry out phase 2 and 3 clinical trials in long-term studies, to evaluate the wound healing activity of plant extracts (e.g., *Croton lechleri* and *Ageratina pichinchensis*).

## Figures and Tables

**Table 1:** Latin American medicinal plants used traditionally for wound healing.

Botanical Family	Plant Name	Plant Part, Way of Administration	Reference
Acanthaceae	*Justicia spicigera* Schltdl.	Infusion of leaves, oral	[8]
	*Ruellia hygrophila* Mart.	Decoction of leaves, topical	[9]
Alismataceae	*Sagittaria montevidensis* Cham. and Schltdl.	Infusion of leaves, oral	[10]
Amaranthaceae	*Achyranthes brasiliana* (L.) Standl. (accepted name: *Alternanthera brasiliana* (L.) Kuntze)	Infusion of aerial parts, oral	[11]
	*Alternanthera pungens* Kunth	Decoction of aerial parts, topical	[9]
	*Chenopodium ambrosioides* L.	Pulverized leaves, topical	[12]
	*Dysphania ambrosioides* (L.) Mosyakin and Clemants	Maceration of leaves, topical	[13]
	*Gomphrena celosioides* Mart.	Cataplasm of leaves, topical	[14]
	*Iresine herbstii* Hook.	Infusion of leaves, topical	[15]
Anacardiaceae	*Anacardium occidentale* L.	Maceration of fruits, topical	[16]
	*Amphipterygium adstringens* (Schltdl.) Standl.	Pulverized bark, topical	[17]
	*Myracrodruon urundeuva* Allemão	Infusion of stem, topical	[18]
	*Schinopsis lorentzii* (Griseb.) Engl.	Bark infusion, topical	[9]
	*Schinus molle* L.	Infusion of leaves, topical	[15]
	*Schinus terebinthifolia* Raddi	Maceration of leaves, topical	[19]
	*Spondias mombin* L.	Resin, topical	[20]
Annonaceae	*Annona squamosa* L.	Pulverized leaves, topical	[21]
Apiaceae	*Cyclospermum leptophyllum* (Pers.) Sprague ex Britton and P. Wilson	Decoction of aerial parts, topical	[14]
	*Eryngium carlinae* F. Delaroche	Infusion of aerial parts, oral	[22]
	*Petroselinum crispum* (Mill.) Fuss	Decoction of whole plant, topical	[23]
Apocynaceae	*Asclepias curassavica* L.	Latex, topical	[24]
	*Asclepias linaria* Cav.	Latex, topical	[25]
	*Cascabela thevetioides* (Kunth) Lippold	Sap, topical	[26]
	*Hancornia speciosa* Gomes	Decoction of roots, topical	[27]
	*Himatanthus drasticus* (Mart.) Plumel	Latex, topical	[28]
	*Plumeria sucuuba* Spruce ex Müll. Arg.	Latex, topical	[29]
	*Prestonia glabrata* Kunth	Infusion of whole plant, topical	[23]
	*Tabernaemontana catharinensis* A. DC.	Bark infusion, oral	[14]
	*Tabernaemontana sananho* Ruiz and Pav.	Bark latex, topical	[30]
	*Vallesia glabra* (Cav.) Link	Decoction of branches, topical	[9]
Aquifoliaceae	*Ilex guayusa* Loes.	Cataplasm of leaves, topical	[31]
Araceae	*Dracontium loretense* K. Krause	Cataplasm of tuber, topical	[32]
	*Monstera lechleriana* Schott	Latex, topical	[30]
	*Synandrospadix vermitoxicus* (Griseb.) Engl.	Tubercle, topical	[33]
Araliaceae	*Oreopanax andreanus* Marchal	Decoction of leaves, topical	[23]
Arecaceae	*Attalea speciosa* Mart.	Seed oil, topical	[29]
	*Socratea exorrhiza* (Mart.) H. Wendl.	Pulverized root, topical	[29]
Aristolochiaceae	*Prosopanche americana* (R. Br.) Baill.	Ashes of whole plant, topical	[33]
Asparagaceae	*Agave atrovirens* Karw. ex Salm-Dyck	Leaves, topical	[34]
Asteraceae	*Acanthospermum australe* (Loefl.) Kuntze	Decoction of leaves, topical	[19]
	*Achillea millefolium* L.	Infusion of whole plant, topical	[22]
	*Ageratina pichinchensis* (Kunth) R.M. King and H. Rob.	Maceration of leaves and root, topical	[35]
	*Baccharis genistelloides* (Lam.) Pers.	Decoction of aerial parts, topical	[12]
	*Baccharis odorata* Kunth	Decoction of leaves, topical	[24]
	*Baccharis salicifolia* (Ruiz and Pav.) Pers.	Decoction of leaves, topical	[24]
	*Bidens pilosa* L.	Infusion of aerial parts, topical	[36]
	*Chaptalia nutans* (L.) Pol.	Infusion of aerial parts, topical	[37]
	*Eupatorium laevigatum* Lam.	Infusion of leaves, topical	[15]
	*Eupatorium subhastatum* Hook. and Arn.	Decoction of aerial parts, topical	[14]
	*Flaveria trinervia* (Spreng.) C. Mohr	Infusion of aerial parts, topical	[17]
	*Galinsoga parviflora* Cav.	Infusion of flowers, oral	[22]
	*Gamochaeta coarctata* (Willd.) Kerguélen	Decoction of whole plant, topical	[38]
	*Grindelia oxylepis* Greene	Infusion of whole plant, topical	[39]
	*Haplopappus venetus* (Kunth) S.F. Blake	Decoction of branches, topical	[39]
	*Heterotheca inuloides* Cass.	Infusion of whole plant, topical	[39]
	*Jungia paniculata* (DC.) A. Gray	Infusion of leaves, topical	[38]
	*Molina latifolia* Ruiz and Pav	Decoction of leaves and bark, topical	[24]
	*Mutisia acuminata* Ruiz and Pav.	Pulverized leaves, topical	[38]
	*Parthenium hysterophorus* L.	Decoction of leaves, topical	[40]
	*Pluchea microcephala* R.K. Godfrey	Decoction of aerial parts, topical	[9]
	*Pluchea sagittalis* (Lam.) Cabrera	Infusion of leaves, topical	[15]
	*Porophyllum ruderale* (Jacq.) Cass.	Pulverized leaves, topical	[30]
	*Psacalium decompositum* (A. Gray) H. Rob. and Brettell	Root infusion, oral	[41]
	*Pseudognaphalium canescens* (DC.) Anderb.	Maceration of leaves and roots, topical	[26]
	*Pseudelephantopus spicatus* (B. Juss. ex Aubl.) C.F. Baker	Cataplasm of aerial parts, topical	[30]
	*Pterocaulon virgatum* (L.) DC.	Decoction of aerial parts, topical	[14]
	*Senecio angulifolius* DC.	Pulverized leaves, topical	[42]
	*Senecio rhizomatus* Rusby	Infusion of leaves, oral	[38]
	*Schkuhria pinnata* (Lam.) Kuntze ex Thell.	Infusion of leaves, oral	[8]
	*Smallanthus sonchifolius* (Poepp.) H. Rob.	Infusion of roots, topical	[23]
	*Solidago chilensis* Meyen	Maceration of leaves, topical	[11]
	*Sphagneticola trilobata* (L.) Pruski	Infusion of aerial parts, topical	[10]
	*Spilanthes alba* L’Hér.	Infusion of aerial parts, topical	[23]
	*Tagetes erecta* L.	Maceration of leaves, topical	[36]
	*Tagetes mandonii* Sch. Bip. ex Klatt	Infusion of leaves, topical	[43]
	*Tagetes terniflora* Kunth	Decoction of leaves and roots, topical	[23]
	*Trixis angustifolia* DC.	Infusion of whole plant, oral	[39]
	*Verbesina crocata* (Cav.) Less.	Infusion of aerial parts, topical	[17]
	*Verbesina scabra* Benth.	Maceration of leaves with oil, topical	[9]
	*Werneria nubigena* Kunth	Infusion of whole plant, topical	[43]
	*Xanthium strumarium* L.	Infusion of whole plant, topical	[15]
Basellaceae	*Anredera diffusa* (Moq.) Sperling	Infusion of leaves, topical	[44]
Begoniaceae	*Begonia glabra* Aubl	Cataplasm of leaves, topical	[30]
Bignoniaceae	*Crescentia cujete* L.	Fruit pulp, oral	[45]
	*Fridericia chica* (Bonpl.) L.G. Lohmann	Cataplasm of leaves, topical	[30]
	*Mussatia hyacinthina* (Standl.) Sandwith	Decoction of bark, oral	[29]
	*Tabebuia serratifolia* (Vahl) G. Nicholson	Cataplasm of bark, topical	[20]
	*Tanaecium nocturnum* (Barb. Rodr.) Bureau and K. Schum.	Decoction of bark, oral	[29]
	*Tecoma stans* (L.) Juss. ex Kunth	Cataplasm of leaves and bark, topical	[46]
Bixaceae	*Bixa orellana* L.	Infusion of stem bark, topical	[47]
Brassicaceae	*Coronopus didymus* (L.) Sm.	Infusion of whole plant, topical	[46]
Burseraceae	*Bursera arida* (Rose) Standl.	Latex, topical	[17]
	*Bursera morelensis* Ramírez	Infusion of bark, oral	[48]
	*Bursera simaruba* (L.) Sarg.	Resin, topical	[37]
Cactaceae	*Cephalocereus senilis* (Haw.) Pfeiff.	Pulp, topical	[26]
	*Echinocereus poselgeri* Lem.	Maceration of roots, topical	[26]
	*Nopalea cochenillifera* (L.) Salm-Dyck	Maceration of roots, topical	[8]
	*Pereskia sacharosa* Griseb.	Infusion of leaves, topical	[47]
Calophyllaceae	*Calophyllum brasiliense* Cambess.	Resin/topical	[45]
Capparaceae	*Capparis osmantha* Diels	Cataplasm of bark, topical	[30]
Caryophyllaceae	*Paronychia chilensis* DC.	Infusion of branches, oral	[46]
Celastraceae	*Maytenus ilicifolia* Mart. ex Reissek	Infusion of whole plant, topical	[49]
	*Maytenus macrocarpa* (Ruiz and Pav.) Briq.	Decoction of bark, topical	[32]
Cleomaceae	*Cleome spinosa* Jacq.	Decoction of roots, topical	[50]
Clusiaceae	*Clusia nemorosa* G. Mey.	Resin, topical	[51]
	*Garcinia macrophylla* Mart.	Resin, topical	[52]
Combretaceae	*Combretum leprosum* Mart.	Infusion of aerial parts, topical	[53]
Commelinaceae	*Commelina coelestis* Willd.	Cataplasm of leaves, topical	[5]
	*Dichorisandra ulei* J.F. Macbr.	Cataplasm of aerial parts, topical	[30]
	*Tradescantia spathacea* Sw.	Cataplasm of leaves, topical	[40]
Convolvulaceae	*Convolvulus crenatifolius* var. peruviana Hallier f.	Decoction of whole plant, oral	[43]
	*Cuscuta grandiflora* Kunth	Decoction of whole plant, topical	[43]
	*Dichondra argentea* Humb. and Bonpl. ex Willd	Decoction of whole plant, topical	[39]
	*Ipomoea asarifolia* (Desr.) Roem. and Schult.	Decoction of aerial parts, topical	[52]
	*Ipomoea batatas* (L.) Lam.	Infusion of leaves, topical	[19]
	*Ipomoea carnea* Jacq.	Pulverized leaves, topical	[9]
	*Ipomoea pes-caprae* (L.) R. Br.	Decoction of whole plant, topical	[52]
	*Operculina hamiltonii* (G. Don) D.F. Austin and Staples	Infusion of roots, topical	[50]
Cordiaceae	*Cordia americana* (L.) Gottschling and J.S. Mill.	Decoction of leaves, topical	[14]
	*Cordia ecalyculata* Vell.	Infusion of leaves, oral	[27]
Crassulaceae	*Sedum dendroideum* DC.	Infusion of leaves, topical	[15]
Cucurbitaceae	*Cucurbita maxima* Duchesne	Sap fruit, topical	[14]
	*Cucurbita pepo* L.	Latex, topical	[40]
Cyatheaceae	*Cyathea cuspidate* Kunze	Decoction of leaves, topical	[54]
Cyperaceae	*Cyperus articulates* L.	Decoction of tubercule, topical	[54]
	*Scirpus californicus* (C.A. Mey.) Steud.	Decoction of the aerial rhizome, oral	[43]
Equisetaceae	*Equisetum bogotense* Kunth	Infusion of leaves, topical	[12]
Euphorbiaceae	*Acalypha monostachya* Cav.	Infusion of leaves, topical	[26]
	*Chamaesyce hirta* (L.) Millsp	Latex, topical	[24]
	*Chamaesyce hypericifolia* (L.) Millsp.	Latex, topical	[24]
	*Croton antisyphiliticus* Mart.	Pulverized roots, topical	[27]
	*Croton draconoides* Müll. Arg.	Resin, topical	[47]
	*Croton lechleri* Müll. Arg.	Resin, topical	[32]
	*Croton mutisianus* Kunth	Latex, topical	[23]
	*Croton pottsii* (Klotzsch) Müll. Arg.	Decoction of whole plant, topical	[39]
	*Croton suaveolens* Torr.	Cataplasm of leaves, topical	[26]
	*Hura crepitans* L.	Infusion of bark, topical	[32]
	*Jatropha curcas* L.	Latex, topical	[45]
	*Jatropha dioica* Sessé ex Cerv.	Infusion of aerial parts, topical	[39]
	*Jatropha gossypiifolia* L.	Latex, topical	[53]
	*Jatropha neopauciflora* Pax	Latex, topical	[17]
	*Machaerium complanatum* Ducke	Cataplasm of bark, topical	[30]
	*Manihot tripartita* (Spreng.) Müll. Arg	Infusion of whole plant, oral	[27]
	*Pedilanthus tithymaloides* (L.) Poit.	Infusion of aerial parts, oral	[36]
	*Sapium haematospermum* Müll. Arg.	Decoction of leaves, topical	[9]
	*Sapium laurifolium* (A. Rich.) Griseb.	Latex, topical	[29]
	*Sapium marmieri* Huber	Latex, topical	[30]
Fabaceae	*Acacia albicorticata* Burkart	Pulverized bark, topical	[33]
	*Acacia aroma* Gillies ex Hook. and Arn.	Decoction of leaves, topical	[33]
	*Acacia caven* Molina	Infusion of bark, topical	[46]
	*Amburana cearensis* (Allemão) A.C. Sm.	Decoction of bark, topical	[50]
	*Bauhinia divaricata* L.	Infusion of leaves, oral	[55]
	*Caesalpinia paraguariensis* (D. Parodi) Burkart	Pulverized bark, topical	[14]
	*Caesalpinia spinosa* (Molina) Kuntze	Decoction of fruit, topical	[24]
	*Cassia tomentosa* L. f.	Pulverized leaves, topical	[38]
	*Copaifera langsdorffii* Desf.	Infusion of shell, oral	[16]
	*Copaifera reticulata* Ducke	Sap, topical	[29]
	*Cercidium praecox* (Ruiz and Pav. ex Hook.) Harms	Ashes of stem and bark, topical	[14]
	*Deguelia rariflora* (Mart. ex Benth.) G.P. Lewis and Acev.-Rodr.	Infusion of roots, oral	[28]
	*Desmodium mexicanum* Sweet	Infusion of whole plant, oral	[12]
	*Erythrina crista-galli* L.	Pulverized bark and leaves, topical	[14]
	*Erythrina dominguezii* Hassl.	Decoction of bark, oral	[29]
	*Erythrina edulis* Triana ex Micheli	Infusion of bark, oral	[23]
	*Geoffroea decorticans* (Gillies ex Hook. and Arn.) Burkart	Pulverized leaves, topical	[14]
	*Gliricidia sepium* (Jacq.) Kunth ex Walp.	Cataplasm of leaves, topical	[37]
	*Indigofera suffruticosa* Mill.	Infusion of leaves, oral	[8]
	*Macrolobium acaciifolium* (Benth.) Benth	Decoction of stem bark, topical	[47]
	*Mimosa albida* Humb. and Bonpl. ex Willd.	Infusion of leaves, oral	[5]
	*Mimosa tenuiflora* (Willd.) Poir.	Maceration of whole plant, topical	[22]
	*Myrocarpus frondosus* Allemão	Maceration of aerial parts, topical	[19]
	*Myroxylon balsamum* (L.) Harms	Infusion of bark, topical	[23]
	*Myroxylon peruiferum* L. f.	Infusion of bark, topical	[46]
	*Parkia multijuga* Benth.	Powdered bark, topical	[31]
	*Peltophorum dubium* (Spreng.) Taub.	Pulverized bark, topical	[49]
	*Poeppigia procera* C. Presl	Decoction of bark, topical	[45]
	*Prosopis juliflora* (Sw.) DC.	Decoction of leaves and seed, topical	[23]
	*Prosopis ruscifolia* Griseb.	Decoction of leaves, topical	[9]
	*Prosopis vinalillo* Stuck.	Decoction of leaves, topical	[9]
	*Psoralea pubescens* Poir	Decoction of aerial parts, topical	[43]
	*Senna morongii* (Britton) H.S. Irwin and Barneby	Decoction of aerial parts, topical	[9]
	*Senna pendula* (Humb. and Bonpl. ex Willd.) H.S. Irwin and Barneby	Ashes of leaves, topical	[33]
	*Stryphnodendron adstringens* (Mart.) Coville	Pulverized bark, topical	[27]
	*Stryphnodendron coriaceum* Benth.	Decoction of bark, topical	[53]
	*Stryphnodendron rotundifolium* Mart.	Infusion of bark, topical	[50]
	*Trifolium amabile* Kunth	Decoction of roots, topical	[43]
	*Vachellia caven* (Molina) Seigler and Ebinger	Pulverized bark, topical	[56]
Geraniaceae	*Geranium mexicanum* Kunth	Pulverized aerial parts, topical	[57]
	*Pelargonium odoratissimum* (L.) L’Hér.	Infusion of leaves, topical	[23]
Heliotropiaceae	*Heliotropium angiospermum* Murray	Decoction of whole plant, topical	[42]
	*Heliotropium curassavicum* var. argentinum I.M. Johnst.	Decoction of aerial parts, topical	[14]
	*Heliotropium procumbens* Mill.	Decoction of aerial parts, topical	[9]
Hypericaceae	*Vismia guianensis* (Aubl.) Choisy	Latex, topical	[58]
Juglandaceae	*Juglans neotropica* Diels	Decoction of leaves, topical	[43]
Lamiaceae	*Leonurus sibiricus* L.	Infusion of leaves, oral	[11]
	*Salvia mexicana* L.	Infusion of leaves, topical	[36]
	*Salvia stachydifolia* Benth	Pulverized leaves, topical	[56]
Lecythidaceae	*Bertholletia excelsa* Bonpl.	Infusion of stem bark, oral	[28]
Loranthaceae	*Struthanthus crassipes* (Oliv.) Eichler	Infusion of leaves, topical	[40]
	*Struthanthus densiflorus* (Benth.) Standl.	Infusion of flowers, oral	[8]
Losaceae	*Mentzelia cordifolia Dombey* ex Urb. and Gilg	Decoction of aerial parts, topical	[38]
Lythraceae	*Cuphea aequipetala* Cav.	Pulverized aerial parts, topical	[59]
Malpighiaceae	*Banisteriopsis caapi* (Spruce ex Griseb.) C.V. Morton	Decoction of stem, topical	[23]
	*Byrsonima sericea* DC	Maceration of bark, topical	[60]
Malvaceae	*Ceiba aesculifolia subsp. parvifolia* (Rose) P.E. Gibbs and Semir	Pulverized bark, topical	[17]
	*Gossypium barbadense* L.	Seeds, oral	[50]
	*Heliocarpus appendiculatus* Turcz.	Latex, topical	[40]
	*Malachra alceifolia* Jacq.	Decoction of whole plant, topical	[37]
	*Modiola caroliniana* (L.) G. Don	Decoction of leaves, topical	[14]
	*Sida rhombifolia* L.	Infusion of roots, oral	[23]
	*Sphaeralcea angustifolia* (Cav.) G. Don	Decoction of leaves, oral	[36]
	*Waltheria communis* A. St.-Hil.	Infusion of whole plant, topical	[15]
Marantaceae	*Maranta arundinacea* L.	Pulverized rhizome, topical	[58]
Meliaceae	*Carapa guianensis* Aubl.	Seed oil, topical	[28]
	*Guarea kunthiana* A. Juss.	Infusion of leaves, topical	[23]
Menispermaceae	*Cissampelos pareira* L.	Pulverized leaves, topical	[14]
Myrsinaceae	*Ardisia compressa* Kunth	Infusion of leaves, topical	[40]
Myrtaceae	*Blepharocalyx tweediei* (Hook. and Arn.) O. Berg	Infusion of leaves, topical	[8]
	*Campomanesia rufa* (O. Berg) Nied.	Infusion of roots, oral	[27]
	*Psidium guajava* L.	Infusion leaves, topical	[57]
	*Psidium guineense* Sw.	Decoction of leaves, topical	[19]
Moraceae	*Dorstenia houstonii* L	Latex, topical	[37]
	*Ficus americana* Aubl.	Resin, topical	[47]
	*Ficus insipida* Willd.	Pulverized bark and leaves, topical	[49]
	*Sorocea houlletiana* Gaudich. (accepted name: *Sorocea guilleminiana* Gaudich.)	Cataplasm of leaves, topical	[30]
Nyctaginaceae	*Mirabilis jalapa* L.	Infusion of leaves, topical	[10]
Onagraceae	*Fuchsia hypoleuca* I.M. Johnst.	Infusion of aerial parts, topical	[23]
	*Fuchsia magellanica* Lam.	Infusion of leaves, topical	[23]
	*Gaura hexandra* Ortega	Decoction of whole plant, topical	[39]
	*Oenothera rosea* L’Hér. ex Aiton	Decoction of whole plant, topical	[8]
Papaveraceae	*Argemone mexicana* L.	Infusion of flowers and seeds, oral	[22]
	*Argemone ochroleuca* Sweet	Decoction of roots, topical	[61]
	*Argemone subfusiformis* G.B. Ownbey	Decoction of aerial parts, topical	[14]
	*Bocconia frutescens* L.	Infusion of leaves, oral	[36]
Petiveriaceae	*Petiveria alliacea* L.	Infusion of aerial parts, topical	[15]
Phytolaccaceae	*Phytolacca icosandra* L.	Infusion of leaves, topical	[51]
Picramniaceae	*Picramnia spruceana* Engl.	Infusion of leaves, topical	[58]
Piperaceae	*Peperomia congona* Sodiro	Infusion of whole plant, oral	[62]
	*Peperomia galioides* Kunth	Pulverized whole plant, topical	[38]
	*Piper aduncum* L.	Infusion of leaves, oral	[8]
	*Piper affictum* Trel.	Cataplasm of leaves, topical	[30]
	*Piper barbatum* Kunth	Infusion of whole plant, topical	[23]
	*Piper confusionis* Trel.	Cataplasm of leaves, topical	[30]
	*Piper dichotomum* Ruiz and Pav	Cataplasm of leaves, topical	[30]
	*Piper ecuadorense* Sodiro	Infusion of leaves, oral	[23]
	*Piper loretoanum* Trel.	Cataplasm of leaves, topical	[30]
	*Piper peltatum* L.	Decoction of leaves, topical	[54]
	*Piper regnellii* (Miq.) C. DC.	Infusion of leaves, topical	[15]
	*Piper sanctum* (Miq.) Schltdl. ex C. DC.	Infusion of leaves, oral	[36]
Plagiochilaceae	*Plagiochila hondurensis* Herzog ex Carl	Cataplasm of whole plant, topical	[31]
Plantaginaceae	*Mecardonia procumbens* (Mill.) Small	Pulverized aerial parts, topical	[59]
	*Plantago tomentosa* Lam.	Decoction of leaves, topical	[10]
	*Scoparia dulcis* L.	Infusion of whole plant, topical	[23]
Poaceae	*Gynerium sagittatum* (Aubl.) P. Beauv.	Pulverized leaves, topical	[29]
Polygonaceae	*Muehlenbeckia tamnifolia* (Kunth) Meisn.	Pulverized leaves, topical	[38]
	*Muehlenbeckia volcanica* (Benth.) Endl.	Infusion of whole plant, oral	[62]
	*Polygonum punctatum* Elliott	Ashes of leaves, topical	[14]
Polypodiaceae	*Campyloneurum fuscosquamatum* Lellinger	Pulverized leaves, topical	[29]
	*Microgramma squamulosa* (Kaulf.) de la Sota	Infusion of whole plant, oral	[46]
Primulaceae	*Cybianthus anthuriophyllus* Pipoly	Cataplasm of leaves and roots	[30]
Pteridaceae	*Adiantum poiretii* Wikstr.	Cataplasm of whole plant, topical	[39]
Rhamnaceae	*Ceanothus azureus* Desf. ex DC	Infusion of whole plant, oral	[5]
Rhizophoraceae	*Rhizophora mangle* L.	Decoction of bark, topical	[45]
Rosaceae	*Rubus glaucus* Benth.	Infusion of leaves, topical	[23]
Rubiaceae	*Carapichea ipecacuanha* (Brot.) L. Andersson	Infusion of roots, topical	[50]
	*Calycophyllum spruceanum* (Benth.) Hook. f. ex K. Schum.	Decoction of bark, topical	[32]
	*Genipa americana* L.	Decoction of fruit, topical	[20]
	*Hamelia patens* Jacq.	Infusion of leaves, oral	[8]
	*Hintonia latiflora* (DC.) Bullock	Pulverized bark, topical	[39]
	*Uncaria guianensis* (Aubl.) J.F. Gmel.	Decoction of bark, oral	[32]
Rutaceae	*Helietta apiculate* Benth.	Infusion of bark, oral	[49]
Salicaceae	*Salix humboldtiana* Willd.	Decoction of bark, topical	[9]
Sapindaceae	*Acer negundo* L.	Sap, topical	[26]
	*Cardiospermum halicacabum* L.	Cataplasm of leaves, topical	[5]
Sapotaceae	*Sideroxylon obtusifolium* (Humb. ex Roem. and Schult.) T.D. Penn.	Infusion of aerial parts, oral	[18]
Saururaceae	*Anemopsis californica* (Nutt.) Hook. and Arn.	Decoction of whole plant, topical	[39]
Scrophulariaceae	*Buddleja cordata* Kunth	Decoction of leaves, topical	[36]
	*Buddleja sessiliflora* Kunth	Infusion of leaves, topical	[55]
Smilacaceae	*Smilax bona-nox* L.	Infusion of roots, oral	[26]
Solanaceae	*Brugmansia candida* Pers.	Maceration of leaves, topical	[36]
	*Brugmansia suaveolens* (Humb. and Bonpl. ex Willd.) Sweet	Maceration of leaves, topical	[15]
	*Capsicum annuum var. glabriusculum* (Dunal) Heiser and Pickersgill	Cataplasm of fruit, topical	[26]
	*Cestrum auriculatum* L’Hér.	Pulverized leaves, topical	[38]
	*Datura ferox* L.	Maceration of leaves with oil, topical	[9]
	*Lycopersicon esculentum* Mill.	Cataplasm of fruit peel, topical	[26]
	*Nicotiana glauca* Graham	Infusion of leaves, topical	[56]
	*Nicotiana tabacum* L.	Infusion of leaves, topical	[11]
	*Physalis chenopodifolia* Lam.	Decoction of flowers, topical	[23]
	*Physalis angulata* L.	Cataplasm of leaves, topical	[32]
	*Solanum erianthum* D. Don	Pulverized whole plant, topical	[19]
	*Solanum glaucophyllum* Desf.	Ashes of stem, topical	[33]
	*Solanum mammosum* L.	Cataplasm of fruit, topical	[32]
	*Solanum nigrescens* M. Martens and Galeotti	Maceration of fruit, topical	[36]
	*Solanum sessiliflorum* Dunal	Infusion of fruit, oral	[32]
Tectariaceae	*Tectaria heracleifolia* (Willd.) Underw.	Infusion of whole plant, oral	[8]
Urticaceae	*Cecropia peltata* L.	Pulverized leaves, topical	[58]
Vernenaceae	*Lippia alba* (Mill.) N.E. Br. ex Britton and Wilson, P.	Infusion of leaves, topical	[19]
	*Lippia gracilis* Schauer	Infusion of leaves, topical	[50]
	*Stachytarpheta cayennensis* (Rich.) Vahl	Maceration of leaves, topical	[11]
	*Verbena hispida* Ruiz and Pav.	Infusion of branches, oral	[46]
	*Verbena litoralis* Kunth	Infusion of whole plant, topical	[23]
Viburnaceae	*Sambucus mexicana* C. Presl ex DC.	Infusion of leaves, oral	[5]
	*Sambucus peruviana* Kunth	Pulverized leaves, topical	[38]
Ximeniaceae	*Ximenia americana* L.	Infusion of bark, topical	[50]
Zamiaceae	*Zamia ulei* Dammer	Pulverized stem, topical	[20]
Zygophyllaceae	*Bulnesia sarmientoi* Lorentz ex Griseb.	Pulverized bark, topical	[63]

## Data Availability

Not applicable.
